# Gene family evolution: an in-depth theoretical and simulation analysis of non-linear birth-death-innovation models

**DOI:** 10.1186/1471-2148-4-32

**Published:** 2004-09-09

**Authors:** Georgy P Karev, Yuri I Wolf, Faina S Berezovskaya, Eugene V Koonin

**Affiliations:** 1 National Center for Biotechnology Information, National Library of Medicine, National Institutes of Health, Bethesda, MD 20894, USA; 2 Department of Mathematics, Howard University, 2400 Sixth Str., Washington D.C., 20059, USA

## Abstract

**Background:**

The size distribution of gene families in a broad range of genomes is well approximated by a generalized Pareto function. Evolution of ensembles of gene families can be described with Birth, Death, and Innovation Models (BDIMs). Analysis of the properties of different versions of BDIMs has the potential of revealing important features of genome evolution.

**Results:**

In this work, we extend our previous analysis of stochastic BDIMs.

In addition to the previously examined rational BDIMs, we introduce potentially more realistic logistic BDIMs, in which birth/death rates are limited for the largest families, and show that their properties are similar to those of models that include no such limitation. We show that the mean time required for the formation of the largest gene families detected in eukaryotic genomes is limited by the mean number of duplications per gene and does not increase indefinitely with the model degree. Instead, this time reaches a minimum value, which corresponds to a non-linear rational BDIM with the degree of approximately 2.7. Even for this BDIM, the mean time of the largest family formation is orders of magnitude greater than any realistic estimates based on the timescale of life's evolution. We employed the embedding chains technique to estimate the expected number of elementary evolutionary events (gene duplications and deletions) preceding the formation of gene families of the observed size and found that the mean number of events exceeds the family size by orders of magnitude, suggesting a highly dynamic process of genome evolution. The variance of the time required for the formation of the largest families was found to be extremely large, with the coefficient of variation >> 1. This indicates that some gene families might grow much faster than the mean rate such that the minimal time required for family formation is more relevant for a realistic representation of genome evolution than the mean time. We determined this minimal time using Monte Carlo simulations of family growth from an ensemble of simultaneously evolving singletons. In these simulations, the time elapsed before the formation of the largest family was much shorter than the estimated mean time and was compatible with the timescale of evolution of eukaryotes.

**Conclusions:**

The analysis of stochastic BDIMs presented here shows that non-linear versions of such models can well approximate not only the size distribution of gene families but also the dynamics of their formation during genome evolution. The fact that only higher degree BDIMs are compatible with the observed characteristics of genome evolution suggests that the growth of gene families is self-accelerating, which might reflect differential selective pressure acting on different genes.

## Background

An extremely broad variety of phenomena in physics, biology, and the social sphere is described by power law distributions. The power laws apply to the distribution of the number of links between documents in the Internet, the population of towns, the number of species that become extinct within a year, the number of sexual and other contacts between people, and numerous other quantities [1-4]. In the field of genomics, the "dominance by a selected few" [5] encapsulated in the power laws applies to the distribution of the number of transcripts per gene, the number of interactions per protein, the number of genes in coexpressed gene sets, the number of genes or pseudogenes in paralogous families, the number of connections per node in metabolic networks, and other quantities that can be obtained by genome analysis [5-9].

Mathematically, these distributions are described by the formula: *P*(*i*) ≈ *ci*^-*γ *^where *P*(*i*) is the frequency of nodes with exactly *i *connections or sets with exactly *i *members, *γ *is a parameter which typically assumes values between 1 and 3, and *c *is a normalization constant. Obviously, in double-logarithmic coordinates, the plot of *P *as a function of *i *is close to a straight line with a negative slope. Recently, it has been shown that the distributions of several genome-related quantities are best described by the so-called generalized Pareto function: *P*(*i*) = *c*(*i *+ *a*)^-*γ *^where *γ *> 0, *a *are parameters [10-13]. At large *i *(*i *>>*a*), this distribution is indistinguishable from a power law, but at small *i*, it deviates substantially, with the magnitude of the deviation depending on *a*.

Power law distributions and the associated scale-free networks are compatible with the intuitively plausible mechanism of evolution by preferential attachment although other modes of evolution are also possible [9,14]. Under preferential attachment, a network or a mathematically analogous object, such as an ensemble of gene families, grows via attachment of new nodes to the pre-existing ones with a probability that is proportional to the degree (number of connections) of the latter.

However, preferential attachment or other general evolutionary principles associated with power law type distributions and scale-free phenomena do not actually explain the emergence of these phenomena in biologically meaningful terms. A biological explanation involves, at a minimum, identifying the elementary events underlying the evolutionary process and the simplest models of evolution that include these events and are compatible with the observations. Under this logic, families of paralogous genes represent a perfect object for evolutionary modeling. Indeed, for these families, elementary evolutionary processes are defined naturally. By definition, paralogous families evolve by gene duplication. It has been long suspected and, with the advent of genomics, established beyond reasonable doubt that genome evolution proceeds largely by duplication of genes or portions thereof, and even long genomic segments or entire genomes [15-20]. All sequenced genomes contain numerous paralogous genes, and in more complex genomes, the majority of genes have at least one paralog [21,22]. Duplication is followed by mutational diversification and gradually leads to functional differentiation of the paralogs. It is thought that such differentiation occurs via the routes of neofunctionalization (emergence, in one of the paralogs, of a new function non-existent in the ancestral gene) [16] and, probably most often, subfunctionalization, i.e., partitioning of subfunctions of the ancestral gene among the paralogs [23,24]. Hence, *duplication *obviously is the first elementary process of genome evolution. Genomes and gene families not only grow but often shrink or, probably most of the time, persist in equilibrium. Therefore, duplication must be counter-balanced by the opposite elementary process, *gene loss*. Again, comparative genomics has shown that gene loss occurs in all species and seems to be extensive in certain lineages, particularly in parasites [25-27]. Finally, genes new to a given lineage may emerge either as a result of a dramatic change after duplication obliterating all "memories" of a gene's origin, or via horizontal gene transfer, or by evolution of a protein-coding gene from a non-coding sequence (rare as this latter process might be). Collectively, the contribution of these processes to genome evolution may be termed *innovation*. Gene duplication, gene loss, and innovation seem to comprise a reasonable minimal set of elementary events for modeling genome evolution. The only potential major addition could be rearrangement of the gene structure whereby genes accrete or lose domains. However, at least for first approximation modeling, these changes could be covered either by duplication, if they do not yield new genes without detectable relationships to pre-existing families, or by innovation if they do. We should further note that evolutionary analysis of paralogous gene families can be reasonably viewed as a study of the evolution of genomes themselves if all genes are viewed as members of paralogous families, ranging in size (number of members) from 1 to *N *(the size of the largest family). Of course, one must keep in mind that describing genome evolution in terms of gene duplication, loss, and innovation represents a high level of abstraction, whereby a gene is considered an atomic unit of evolution, and mutation processes occurring within a gene are ignored. However, numerous comparative-genomic studies have shown the utility of the gene-level abstraction both for systematic prediction of the functions of uncharacterized genes using the patterns of their distribution in diverse genomes [28-31] and for understanding general evolutionary trends. A striking recent example of the latter type of achievement is the demonstration that different functional categories of genes scale differently with genome size, with the steepest ascent of regulatory genes offering a plausible explanation for the observed limits of genome size in prokaryotes [32].

A natural framework for modeling evolution of gene families is a birth-and-death process, a concept well explored in many physical and chemical contexts [33]. Duplication constitutes a gene birth, and gene loss is a death event; innovation also can be readily incorporated in this context. The birth-and-death approach has been applied to modeling the evolution of paralogous genome family sizes [6,12,34], the distribution of folds and families in the entire protein universe [35], and protein-protein interaction networks [36,37]. For over a century since the publication of Darwin's seminal work [38], biologists believed that evolution at all levels is largely driven by natural selection [39]. However, the advent of molecular evolution shifted the perspective by demonstrating, largely through the work of Kimura and his school, that many, if not most, of the fixed nucleotide substitutions are effectively neutral [40]. Recent comparative analyses of gene expression led to the expansion of the neutral evolution concept beyond the genome sequence, at least to the level of the transcriptome [41,42]. Perhaps the principal importance of the neutral theory is that it leads to a change of the prevailing null hypothesis of evolutionary biology: neutrality should be taken as the null hypothesis, and selection should be invoked only when this hypothesis can be rejected. Birth and death models naturally fit this paradigm because they do not include the notion of selection (at least not explicitly). It is therefore of considerable interest to determine whether or not simple models of this class can be rejected as the explanation for various observed features of genomes.

In the previous work [12], we examined in detail simple *deterministic *models of genome evolution, which we dubbed BDIMs, after birth (duplication), death (elimination), and innovation (*de novo *emergence or acquisition via horizontal gene transfer) models. We showed that the power law asymptotic of the size distribution of gene families appears if, and only if, birth and death rates of domains in families of sufficiently large size are balanced (asymptotically equal up to the second order) and that any power asymptotic with *γ *≠ 1 appears only if the per gene birth/death rates depend on the size of the gene family. We showed that the simplest model that adequately approximates the empirical data on gene (domain) family size distributions is the *linear *2^nd ^order balanced BDIM.

Subsequently, we expanded the BDIM framework by introducing stochastic BDIMs, which account not only for the stationary state of the gene ensemble but also for the characteristics of evolution of the system, such as the probability of the formation of a family of the given size before extinction and the mean times of formation and extinction of a family of a given size [43]. We first investigated these issues for the linear 2^nd ^order balanced stochastic BDIM. Given the published estimates of the rates of gene duplication and loss [24], we found that this version of BDIM, which gives a good approximation of the stationary distributions of family sizes for different genomes, predicts completely unrealistic mean times for reaching the observed sizes of the largest domain families. In computer simulations with a large ensemble of genes, even the minimum time required for the formation of the largest family was shown to be unrealistically long. Thus, the linear BDIM is incompatible with the estimates of the rate of genome size growth derived from the empirical data. Therefore we performed a preliminary examination of non-linear, higher degree BDIMs and showed that the rate of genome size growth increases with the degree of the model, rendering non-linear BDIMs more realistic models of genome evolution [43].

Here, we present a detailed analysis of the properties of different non-linear stochastic BDIMs, including polynomial, rational, and logistic ones, which were obtained by the appropriate transformations of the original linear model. These models generated the same stationary family size distribution, but the stochastic properties of the higher order models were dramatically different from those of the linear BDIM. The mean number of elementary events, duplications and deletions, which are required for the formation of the largest family, decrease monotonically with the increase of the model degree. By contrast, the mean time of formation of a gene family of the given size under a fixed average duplication rate went through a minimum depending on the model degree; typically, the model degree corresponding to this minimum was between 2 and 3. However, even with this optimal degree, the mean times of formation of the largest families in different genomes were unrealistically long.

The times of formation and extinction of gene families are random variables with unknown distributions. Therefore it was important to determine the variance of these times and the number of elementary events preceding the formation and extinction of the largest families. We found that the coefficients of variation were very large such that the extreme values of the formation times for the largest family could differ from the mean time by at least two orders of magnitude. Thus, for assessing the feasibility of the formation of the largest families under a given model, the relevant value is not the mean but the minimal time of family formation over the entire ensemble of genes. Using Monte Carlo simulations, we show that the minimal time required for the formation of families of the expected size under BDIMs of the orders between 2–3 is compatible with the timescale of genome evolution.

## Results and discussion

### 1. Definitions and empirical data

#### The basic BDIM definitions and assumptions

We treat a genome as a "bag" of genes (or, more precisely, portions of genes) encoding protein domains (or simply *domains *for brevity; see [12] for details). Domains are treated as independent evolving units disregarding co-occurrence of domains in multidomain proteins. Each domain is considered to be a member of a family, which may have one or more members. In this work, we interchangeably refer to domain families or gene families. Three types of elementary events are postulated: i) *b*i*rth*, which yields a new member in the same domain family as a result of gene duplication, ii) *death*, i.e., inactivation and/or deletion of a domain, and iii) *innovation*, which generates a new, single-member family. Innovation may occur via domain evolution from a non-coding sequence or a non-globular protein sequence, via horizontal gene transfer from another species, or via radical change of a domain after duplication. The rates of elementary events are defined as the probabilities of the respective events during an infinitesimally short time interval [44] and is postulated to be independent of time (all analyzed models are homogeneous) and of the structure, biological function, and other features of individual domain families. Clearly, these assumptions are simplifications made in order to avoid prohibitively complex models; the justification is that, over large (genome-wide) ensembles of families and long time intervals, the existing non-homogeneities are likely to cancel out, making homogeneous models realistic. It may be useful to emphasize that homogeneity of the models does not imply constancy of the number of events during any finite time interval, which is a random variable.

The data on the size of domain families in sequenced genomes were obtained as described previously [12]. Briefly, the domains were identified by comparing the CDD library of position-specific scoring matrices (PSSMs) for domains extracted from the Pfam and SMART databases, to the protein sequences from completely sequenced eukaryotic and prokaryotic genomes  using the RPS-BLAST program [45].

In a finite genome, the maximum number of domains in a family cannot exceed the total number of domains and, in reality, is probably much smaller. Let *N *be the maximum possible number of domain family members (this limit is introduced for technical reasons; however, this should not be perceived as a biologically unrealistic assumption because *N *can be made extremely large, e.g., to exceed the number of genes in the largest known genome by several orders of magnitude; furthermore, almost all of the results below are valid with *N *= ∞ under certain well defined conditions, which ensure the existence of the ergodic distribution of the birth-and-death process). We also consider virtual, "empty" families that consist of 0 domains. In the stochastic BDIMs, newborn domains are extracted from this class and dead domains return to it. Originally, we examined exclusively the deterministic version of the BDIMs [12]. Introduction of the 0 class "closes" the model and allows us to transform it into a Markov process, which provides for the possibility to explore the stochastic properties of the system [43]. In these stochastic models, innovation was not introduced explicitly as it was in the deterministic models, but was implied in the emergence of domains from the 0 class.

Let *p*_*i*_(*t*) be the frequency of a domain family of size *i*. Then *p*_*i*_(*t*) satisfy a system of forward Kolmogorov equations for birth-and-death process (e.g., [44,46]):

d*p*_0_(*t*)/d*t *= -*λ*_0_*p*_0_(*t*) + *δ*_1_*p*_1_(*t*),

d*p*_*i*_(*t*)/d*t *= *λ*_*i*-1_*p*_*i*-1_(*t*) - (*λ*_*i *_+ *δ*_*i*_)*p*_*i*_(*t*) + *δ*_*i*+1_*p*_*i*+1_(*t*) for 0 <*i *<*N*,     (1.1)

d*p*_*N*_(*t*)/d*t *= *λ*_*N*-1_*p*_*N*-1_(*t*) - *δ*_*N*_*p*_*N*_(*t*).

Mathematically, (1.1) defines the state probabilities of a birth-and-death process with the finite number of states {0,1,...*N*} and reflecting boundaries in 0 and *N*. The evolution of individual trajectories of the birth-and-death process *X*(*t*), whose state probabilities satisfy the system (1.1), can be described as follows. At the starting time, the system is situated in some initial state *x*_0_. The time axis {*t *≥ 0} can be divided into intervals [0,*τ*_1_), [*τ*_1_, *τ*_2_), [*τ*_2_, *τ*_3_) ... such that *X*(*t*) is a constant on each interval. If, at the moment *τ*_*n*_, the system was situated in the point *x*_*n *_= *i*, then, in the moment *τ*_*n*+1_, the system moves either into the state *i*+1 with the probability *β*_*i *_= *λ*_*i*_/(*λ*_*i *_+ *δ*_*i*_) or into the state *i*-1 with the probability *μ*_*i *_= *δ*_*i*_/(*λ*_*i *_+ *δ *_*i*_). The *sojourn time t*_*i *_= *τ*_*n*+1 _- *τ*_*n *_between the arrival at the point *x*_*n *_= *i *and the exit from this point is a random variable independent of the previous history of the system and is distributed exponentially: P{*t*_*i *_≥ *x*} = exp(-(*λ*_*i *_+ *δ*_*i*_)*x*). Note that the random variables *t*_*i *_are independent, and the mean sojourn time, *E*(*t*_*i*_), in the state *i *is *E*(*t*_*i*_) = 1/(*λ*_*i *_+ *δ*_*i*_).

Process (1.1) has a unique stationary ergodic distribution *p*_0_,...,*p*_*N *_defined by the equalities d*p*_*i*_(*t*)/d*t *= 0 for 0 ≤ *i *≤ *N*. Let *J*(*i, t*) = *δ*_*i*_*p*_*i*_(*t*) - *λ*_*i*-1_*p*_*i*-1_(*t*) be the *current *through the state *i *in *t *time moment, *J*(*i*) = *δ*_*i*_*p*_*i *_- *λ*_*i*-1_*p*_*i*-1 _be the current in the stationary state. Then the equation for the stationary distribution can be written as *J*(*i*+1) - *J*(*i*) = 0. As the system is closed, *J*(0) = 0 and hence *J*(*i*) = 0 for all *i*, such that

*p*_*i *_/ *p*_*i*-1 _= *λ*_*i*-1_/*δ*_*i*_.





We will consider also the variant of this model with states {1,...*N*} and reflecting boundaries in states 1 and *N*:

d*p*_1_(*t*)/d*t *= -*λ*_1_*p*_1_(*t*) + *δ*_2_*p*_2_(*t*),

d*p*_*i*_(*t*)/d*t *= *λ*_*i*-1_*p*_*i*-1_(*t*) - (*λ*_*i *_+ *δ*_*i*_)*p*_*i*_(*t*) + *δ*_*i*+1_*p*_*i*+1_(*t*) for 1 <*i *<*N*,     (1.3)

d*p*_*N*_(*t*)/d*t *= *λ*_*N*-1_*p*_*N*-1_(*t*) - *δ*_*N*_*p*_*N*_(*t*).

This model describes the evolution of the size of a domain family that includes an indispensable (essential) gene and is not allowed to go extinct. Similarly, for model (1.3), the ergodic distribution is:





The ergodic distribution (1.2) (or 1.4) is globally stable and is approached exponentially with respect to time from any initial state. The asymptotic of the ergodic distribution is completely defined by the asymptotic behavior of the function *χ*(*i*) ≡ *λ*_*i*-1_/*δ*_*i*_. Let us suppose that, for large *i*, the following expansion is valid:

*χ*(*i*) ≡ *λ*_*i*-1_/*δ*_*i *_= *i*^*s *^*θ *(1-*γ*/*i *+ *O*(1/*i*^2^))     (1.5)

Then, the asymptotical behavior of the stationary distribution of model (1.1) is completely defined by three parameters: *s*, *θ *and *γ *([12]). In particular, if the birth-and-death process is the 1^st ^order *balanced*, i.e. if, by definition, *s *= 0 in (1.5), then, asymptotically, *p*_*i *_~ *θ*^*i*^*i*^-*γ *^. If the process is 2^nd ^order balanced, i.e. *s *= 0 and *θ *= 1, then *p*_*i *_~ *i*^-*γ*^.

The complete description of all possible asymptotics of the ergodic distributions of model (1.1) under condition (1.5) is given in Mathematical Appendix, Theorem 1 (hereinafter all references of the form (A.m.n) refer to the corresponding formula in the Mathematical Appendix [see Additional file 1]). It asserts that a large class of models, namely the second order balanced BDIMs, provide any given power asymptotic of the stationary frequency distributions of family sizes.

### 2. Classification of BDIMs

#### Linear BDIM

The simplest model that shows the generalized Pareto distribution is the *linear *BDIM with

*λ*_*i *_= *λ*(*i+a*), *δ*_*i *_= *δ*(*i *+ *b*) for *i *> 0, *λ*, *δ*, *a *and *b *are constants.     (2.1)

The equilibrium distribution of domain family sizes is defined by:



So, if *λ *= *δ *(*θ *= 1), the resulting 2^nd ^order balanced linear BDIM has a power asymptotics with *γ *= 1 + *b *- *a*.

#### Polynomial BDIM

Informally, polynomial BDIMs can be introduced as follows. Under the linear BDIM, the dependence of the birth and death rates on family size is very weak; although each gene "senses" the size of the family (as reflected in the non-zero parameters *a *and *b*), this dependence cannot be interpreted as a specific form of interaction between family members. If such interactions are postulated, *λ*_*i *_~ *P*_*n*_(*i*) and/or *δ*_*i *_~ *Q*_*m*_(*i*), where *P*_*n*_(*i*), *Q*_*m*_(*i*) are polynomials on *i *of the *n*-th and *m*-th degrees. The ergodic distribution of the stochastic polynomial BDIM of the form (1.1) and (1.3) is asymptotically the same as that of the originally described deterministic polynomial BDIM [12], see Appendix (A.1.4), (A.1.5) [see Additional file 1] and Proposition 2 for details. We show here that non-linear polynomial 2^nd ^order balanced BDIM predict evolution rates that are dramatically greater than those for the linear BDIM.

As an example, let us consider the quadratic BDIM in more detail. It takes into account the simplest, pairwise interaction between family members, which leads to *λ*_*i *_~ *i*^2 ^and/or *δ*_*i *_~ *i*^2^, i.e., one or both rates are polynomials on *i *of the second degree. If the polynomial degrees of the birth and death rates are different (e.g., *λ*_*i *_~ *i *and *δ*_*i *_~ *i*^2^), the corresponding BDIM is non-balanced, and equilibrium frequencies have no power asymptotics. Thus, let

*λ*_*i *_= *λ *(*i*^2 ^+ *r*_1_*i *+ *r*_2_), *δ*_*i *_= *δ*(*i*^2 ^+ *q*_1_*i *+ *q*_2_),     (2.3)

where *λ*, *δ*, *r*_*k*_, *q*_*k*_, *k *= 1,2 are constants (such that *λ*_*i*_, *δ*_*i *_are positive for all *i*); equivalently,

*λ*_*i *_= *λ *(*i *+ *a*)(*i *+ *a*_2_), *δ*_*i *_= *δ *(*i *+ *b*)(*i *+ *b*_2_).

Then, *r*_1 _= *a *+ *a*_2_, *q*_1 _= *b *+ *b*_2_, and

*χ*(*i*) = *λ*_*i*-1_/*δ*_*i *_= *θ *(1 + (*r*_1 _- *q*_1 _- 2)/*i *+ *O*(1/*i*^2^)), where *θ *= *λ*/*δ*.

The quadratic BDIM has equilibrium sizes of domain families (see A.1.6)

*p*_*i *_≈ *c*_2_*p*_0 _*λ*_0_/*λθ*^*i*^*i*^*ρ*-2^

where *ρ *= *r*_1 _- *q*_1_, *c*_2 _= *p*_0 _[(Γ (1 + *b*) Γ (1 + *b*_2_)] / [Γ(1 + *a*) Γ (1 + *a*_2_)], and



Thus, if the quadratic BDIM is 2^nd ^order balanced, then *p*_*i *_~ *i*^*ρ*-2^. Note that the asymptotic behavior frequencies *p*_*i *_do not depend on free coefficients *r*_2 _and *q*_2 _in (2.3), but only on *θ *and *r*_1 _- *q*_1_, although the constant *c*_2 _could depend on the free coefficients *r*_2_, *q*_2_.

#### Rational BDIM

Rational models comprise a rather general class of BDIMs, for which the asymptotic behavior of the equilibrium frequencies and equilibrium sizes of domain families are fully tractable. The ergodic distribution of the stochastic rational BDIM is asymptotically the same as that of the deterministic rational BDIM [12]. In particular, if the model is 2^nd ^order balanced, then *p*_*i *_~ *i*^-*γ*^, (see A.1.2 and Proposition 1 in the Appendix for details [see Additional file 1]).

**Figure 1:**
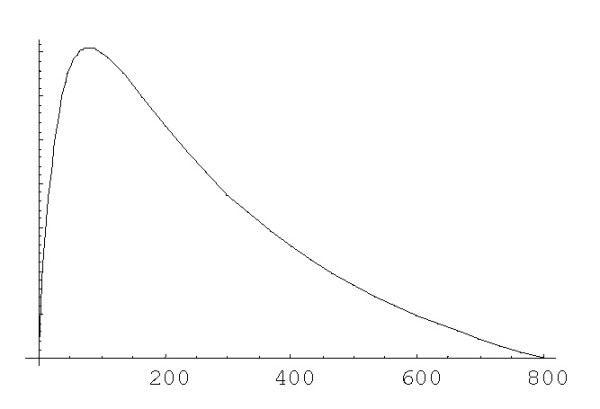
Dependence of the birth rate (*λ*_*i *_= (*i *+ *c*_1_)/(*i *+ *c*_2_)^2^) on the family size.

The rational BDIMs can describe a substantially wider class of birth and death rates compared to polynomial models. In particular, birth rate can have a maximum at some specific value of family size and then decrease with further growth of the size, e.g., as shown in Fig. 1. This dependence of rates on family size can be described by the rational model with *λ*_*i *_= *λ*(*i *+ *a*_1_)/(*i *+ *a*_2_)^2^, *δ*_*i *_= *δ*(*i *+ *b*_1_)/(*i *+ *b*_2_)^2^.

#### Logistic BDIM

Evidently, the number of size classes of protein families, *N*, should be finite, although intrinsic features that could determine the value of *N *so far have not been considered (the impossibility of an infinite genome is self-evident but one would expect a much tighter bound based, e.g., on the limited time and resources available for genome replication and expression). Under the BDIMs described above, birth rate grows monotonically as the family size increases from 1 to *N *and then abruptly drops to 0 (since families of size *N*+1 or greater are not allowed). However, this behavior is an arbitrary simplification of the model and hardly can reflect the actual process of genome evolution.

In population dynamics models, the finiteness of a population size typically results from the "saturation type" of growth: the growth rate tends to 0 as the population size tends to the maximal possible value (see, e.g., [47]). It seems likely that, during genome evolution, gene duplication (and death) rate also tends to 0 as duplications leading to increase in gene number become deleterious when the size of some paralogous families becomes prohibitively large. The simplest formalism, which yields this type of population growth, is the logistic form of the birth rate. Logistic-like stochastic models have been investigated in various applications (e.g., [48,49]), which considered a birth-and-death process with the rates

*λ *(*i*) = *c*_3_(*c*_1 _+ *i*)(*N-i*), *δ*(*i*) = *c*_3_*i*(*c*_2_*-i*), *c*_*k *_> 0, *k *= 1,2,3, *c*_2 _>*N*.

This model produces log-normal and log-series distributions; with the appropriate values of parameters, power low distributions of frequencies also appear, but only for intermediate values of *i*, namely, 1 <<*i *<<*N *and *N *>> 1.

#### Non-linear transformation of BDIM

We have shown previously [43] that the following modification of any form of BDIM:

λ*_i _= *λ*_*i*_*g*(*i*), δ*_i _= *δ*_*i*_*g*(*i*-1)     (2.4)

where *g*(*i*), *i *= 0,...*N*, is a positive function, *g*(0) = 1, results in a BDIM with the same ergodic distribution of the family sizes as the original one. In particular, modifications of a linear BDIM with g(*i*) = (*i *+ 1)^*d*-1 ^or g(*i*) = (*i *+ 1)^*d*-1^(1 - *i*/(*N *+ *c*)) define, respectively, wide classes of rational or logistic BDIMs with the same stationary distribution as the original linear BDIM, but with manifestly different dynamic properties.

### 3. Probability of formation of a family of the given size before extinction and mean and variance of extinction time

In is known [44] that the probability for the birth-and-death process to reach state *n *before reaching state 0 from an initial state *i*> 0 is given by formula (A.2.2). In terms of BDIM (1.1), this means that the probability of formation of a family of size *n *starting from a family of size *i *before getting to extinction is given by (A.2.2).

The random birth-and-death process (1.1) certainly visits state 0 in the course of time; this means that any domain family will eventually get extinct (and then, formally, can be "reborn", returning from the 0-class). Below we compute the mean time to extinction of a family of the given size for different versions of BDIM; the mean time to extinction of the largest family in the given genome is of particular interest.

Let us denote *S*(*n*)=inf{*t*:*X*(*t*) = 0|*X*(0)=*n*} the time to the first passage of state 0 from the initial state *n*; *S*(*n*) is a random variable for each *n*. The mean time to extinction of the family of initial size *n, E*(*S*(*n*)), is given by the general formula A.3.2.

#### Linear BDIM

We have shown previously that, for the linear 2^nd ^order balanced BDIM, the probability that a singleton expands to a family of size *n *before dying, *P*^(1)^(1,*n*) has the power asymptotics for large *n *(A.2.5). The values of probabilities *P*^(1)^(1,*n*) for different species are shown in Table 1; these probabilities are no greater than ~10^-4 ^- 10^-5^. The mean time to extinction, *E*(*S*(*n*)), can be calculated using the relation *E*(*S*(*n*)) = 1/*λE*^(1)^_*n*_, where *E*^(1)^_*n*_, the mean time to extinction expressed in the 1/*λ *time units, is given by formula A.3.3 (see Table 1 for some numerical data and Figs. 1,2 in [43]).

**Table 1:** Family evolution under the linear BDIM (*d*=1)

	*N*	P^(d)^(1,N) *10^2^	*e^(d)^_N_*	*E^(d)^_N_*	*f^(d)^_N_*	*M^(d)^_N_*	*M^(d)^_N_/E^(d)^_N_*	*c^(d)^_du_*	*T^(d)^_N_*
Sce	130	0.284	295267	47.46	260080	20381.6	429.5	1.903	1939.3
Dme	335	0.227	778830	153.74	734725	37409.9	243.3	1.784	3337.0
Cel	662	0.160	1.866*10^6^	347.76	1.803*10^6^	68709.6	197.6	1.523	5232.2
Ath	1535	0.016	2.150*10^7^	702.65	2.087*10^7^	529639.	753.8	2.382	63080.0
Hsa	1151	0.026	1.329*10^7^	505.26	1.29*10^7^	300665.	595.1	2.721	40905.5
Tma	97	0.060	681356	31.47	513450	80677.3	2563.6	1.109	4473.6
Mth	43	1.125	37131.5	14.91	28570	4707.04	315.9	1.091	256.8
Sso	81	0.461	129115	30.14	98440	12853.5	426.5	1.253	805.3
Bsu	124	0.284	237343	48.89	202150	22921.0	468.8	1.320	1512.8
Eco	140	0.155	440665	51.67	375943	37959.8	734.7	1.544	2930.5

For the linear BDIM (*d *= 1) and for the largest family of size *N *in each genome, the table shows the probability of formation *P*^(*d*)^(1,*N*), mean number of events before extinction of the largest family *e*^(*d*)^_*N*_; mean number of events before formation of the largest family from a singleton, *f*^(*d*)^_*N*_; mean times of formation *M*^(*d*)^_*N *_and extinction *E*^(*d*)^_*N *_(in 1/*λ *units); the value of coefficient *c*^(*d*)^_*du *_= *r*_*du*_/*λ*; mean times of formation *T*^(*d*)^_*N *_in Ga (10^9 ^yrs) under *r*_*du *_= 2 × 10^-8^. The model parameters were genome-specific as determined previously [12]. and were the same for all model degrees according to (2.4). Species abbreviations: Sce, *Saccharomyces cerevisiae*, Dme, *Drosophila melanogaster*, Cel, *Caenorhabditis elegans*, Ath, *Arabidopsis thaliana*, Hsa, *Homo sapiens*, Tma, *Thermotoga maritima*, Mth, *Methanothermobacter thermoautotrophicum*, Sso, *Sulfolobus solfataricus*, Bsu, *Bacillus subtilis*, Eco, *Escherichia coli*.

**Figure 2:**
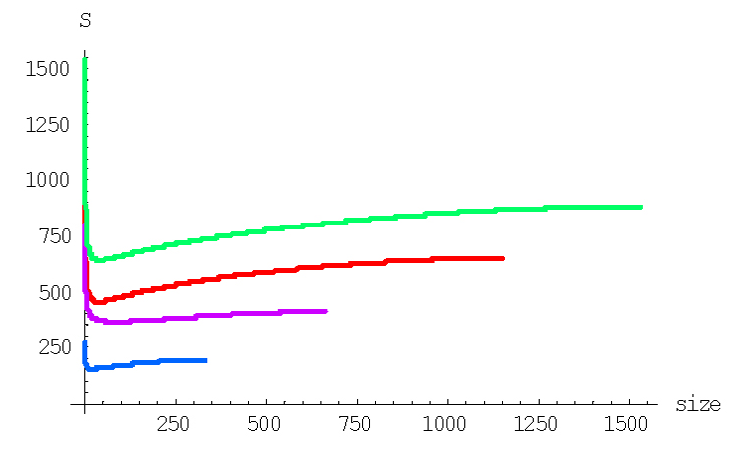
Coefficient of variation of the extinction time versus the family size for the linear BDIM. The model parameters are for *D. melanogaster *(blue), *C. elegans *(purple), *H. sapiens *(red), *A. thaliana *(green) (Table 1 in [43]).

The variance of extinction time *Var*(*S*(*n*)) for the linear 2^nd ^order balanced BDIM is *Var*(*S*(*n*)) = 1/*λ*^2^*W*^(1)^_*n*_, where *W*^(1)^_*n *_can be calculated using the formula (A.3.7). The plot of the coefficient of variation *s*^(1)^_*n *_= (*W*^(1)^_*n*_)^1/2^/*E*^(1)^_*n *_versus *n *for different species is shown in Fig. 2 (see also Table 1 for some numerical data). Clearly, the extinction time can vary within an extremely broad range of values. 

#### Non-linear polynomial and rational BDIM

The stochastic behavior of the system and its characteristics also can be investigated within the broader framework of rational BDIMs. We will examine models represented as transformed linear BDIM (2.1), with

*λ*_*i *_= *λ*(*i *+ *a*)(*i *+ 1)^*d*-1^, *δ*_*i *_= *λ*(*i *+ *b*)*i*^*d*-1^,     (3.1)

where *d *≥ 1 is the model degree. Let us recall that Theorem 1 (Mathematical Appendix [see Additional file 1 ]) shows that the highest degrees and the corresponding coefficients of the birth and death rates at *i*^*d *^must be equal to provide for the power asymptotics of the stationary distribution, *P*(*i*) ~ *i*^-*γ*^. The power *γ *of this distribution is completely determined by the degree *d *and the coefficients at *i*^*d*-1^. Thus, the model (1.1), (3.1) is representative of all rational BDIMs of the degree *d *with a given power asymptotic (*γ *= *b *- *a *+ 1) of the stationary distribution. Besides, according to Proposition 1, this distribution for model (3.1) is exactly the same as for the corresponding linear model with *λ*_*i *_= *λ *(*i *+ *a*), *δ*_*i *_= *λ *(*i *+ *b*), which was studied in detail in [12].

**Figure 3:**
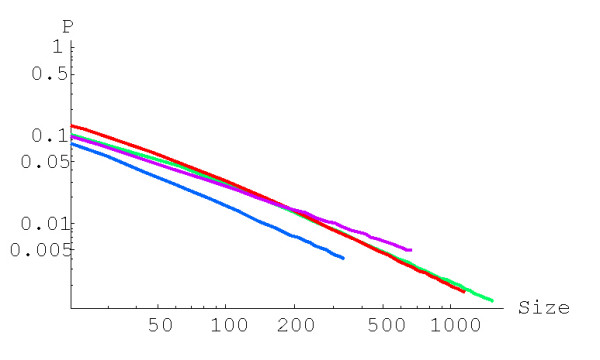
Probability of family formation starting from a singleton, *P*^(2)^(1,*n*), versus the family size (*n*) for the quadratic BDIM (in double logarithmic scale). The model parameters are for *D. melanogaster *(blue), *C. elegans *(purple), *H. Sapiens *(red), *Arabidopsis thaliana *(green).

**Figure 4:**
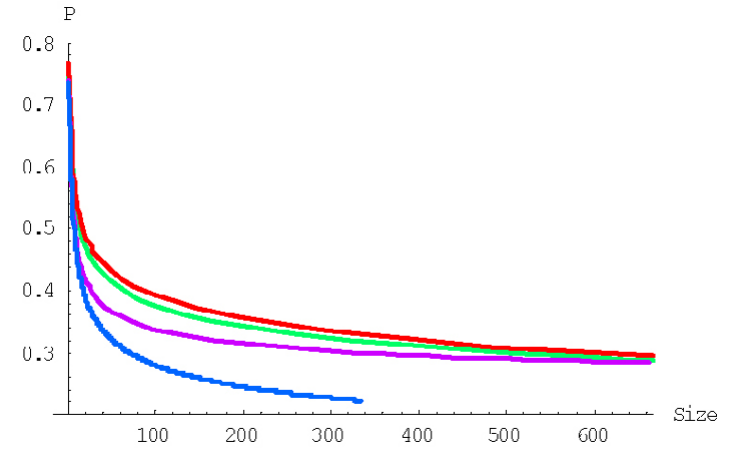
Probability of family formation from a singleton, *P*^(3)^(1,*n*), versus the family size (*n*) for the cubic BDIM. The model parameters are for *D. melanogaster *(blue), *C. elegans *(purple), *H. Sapiens *(red), *Arabidopsis thaliana *(green).

**Figure 5:**
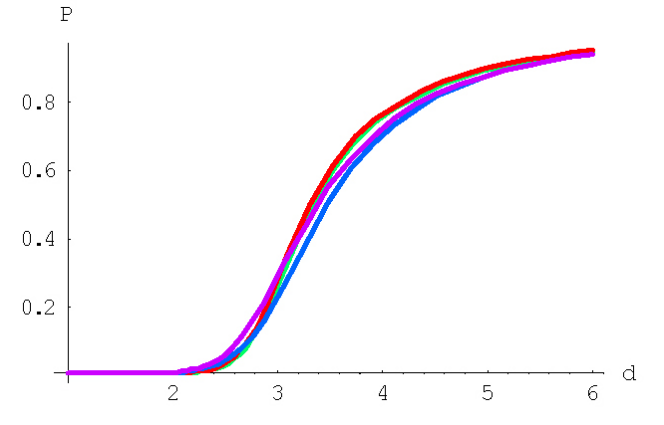
Probability of formation of the largest family starting from a singleton, *P*^(*d*)^(1,*N*), for rational BDIMs depending on the model degree *d*. The model parameters are for *D. melanogaster *(blue), *C. elegans *(purple), *H. Sapiens *(red), *Arabidopsis thaliana *(green).

We applied formula (A.2.6) 



with *g*(*i*) = (*i *+ 1)^*d*-1^, to calculate the probability of formation of a family of the given size from a singleton before getting to extinction for the BDIM of degree *d, P*^(*d*)^(1,*n*). For example, the probabilities *P*^(2)^(1,*n*) and *P*^(3)^(1,*n*) for the quadratic and cubic BDIMs, respectively, are given by this formula with g(*i*) = *i *+ 1 and g(*i*) = (*i *+ 1)^2^, respectively. Figures 3 and 4 show the dependence of the probabilities *P*^(2)^(1,*n*) and *P*^(3)^(1,*n*) on the family size *n *for different species. The dependence of the probability *P*^(*d*)^(1,*N*) of the formation of the largest family on the model degree is shown in Fig. 5.

The mean time to extinction for the rational BDIM (1.1), (3.1) with a fixed *d *is calculated using the formula (A.3.4)  where 



**Figure 6:**
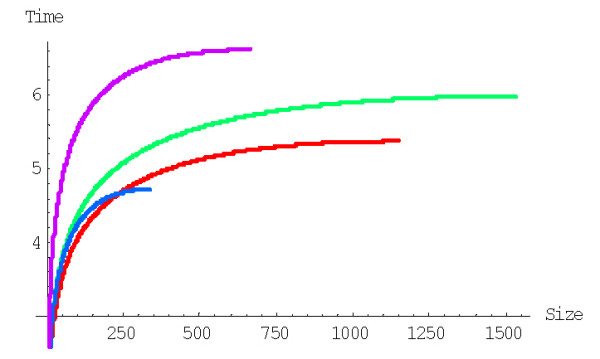
Mean time to extinction (in 1/*λ *units) depending on the family size for the quadratic BDIM. The model parameters are for *D. melanogaster *(blue), *C. elegans *(purple), *H. Sapiens *(red), *Arabidopsis thaliana *(green).

**Figure 7:**
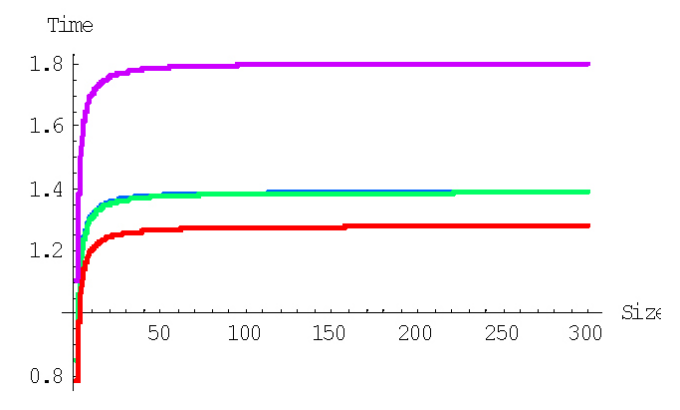
Mean time to extinction (in 1/*λ *units) depending on the family size for the cubic BDIM. The model parameters are for *D. melanogaster *(blue), *C. elegans *(purple), *H. Sapiens *(red), *Arabidopsis thaliana *(green).

**Figure 8:**
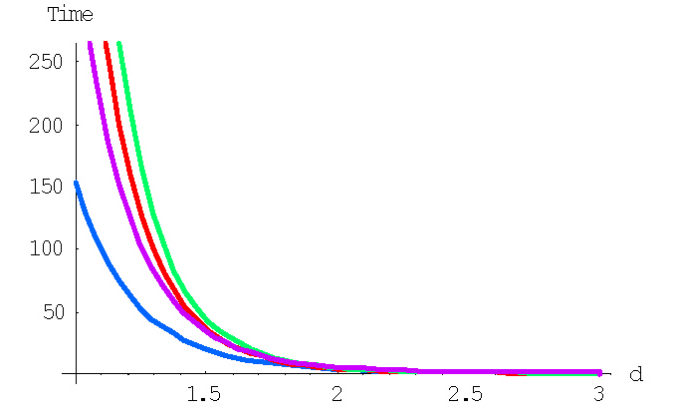
Mean time to extinction (in 1/*λ *units) of the largest family for the rational BDIM depending on the model degree *d*. The model parameters are for *D. melanogaster *(blue), *C. elegans *(purple), *H. Sapiens *(red), *Arabidopsis thaliana *(green).

**Figure 9:**
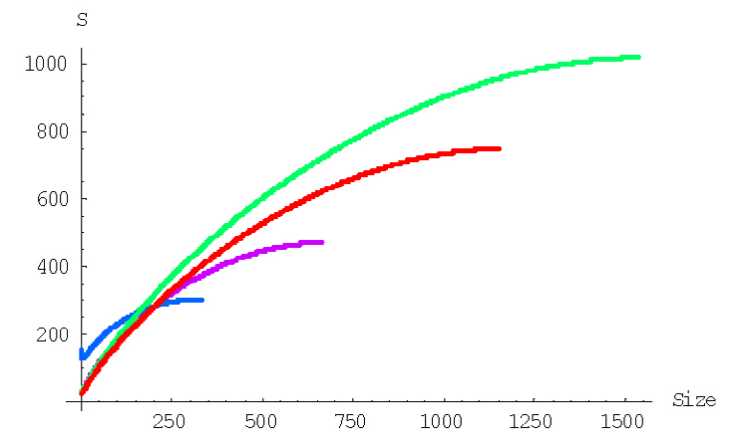
Coefficient of variation of the time to extinction depending on the family size for the quadratic BDIM. The model parameters are for *D. melanogaster *(blue), *C. elegans *(purple), *H. Sapiens *(red), *Arabidopsis thaliana *(green).

**Figure 10:**
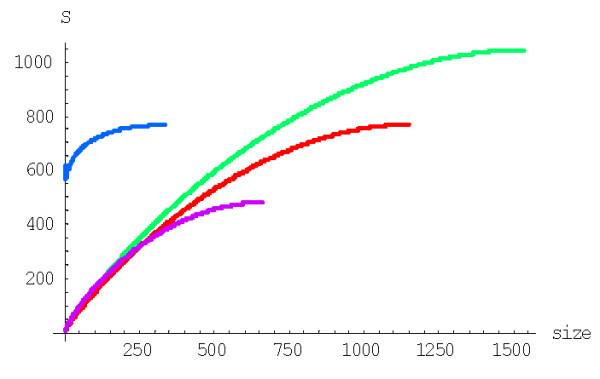
The coefficient of variation of extinction time versus family size for the cubic BDIM. The model parameters are for *D. melanogaster *(blue), *C. elegans *(purple), *H. Sapiens *(red), *Arabidopsis thaliana *(green).

Here E^*^_n _is the mean time to extinction in the 1/*λ *time units. Figures 6 and 7 show the dependence of *E*^(2)^_*n *_and *E*^(3)^_*n *_on *n *for the quadratic and cubic BDIMs, respectively. Fig. 8 shows the mean times of extinction of the largest family, *E*^(*d*)^_*N*_, for different species, depending on the model degree *d*. Some numerical values of the mean time to extinction for quadratic and cubic BDIMs and different species are given in Tables 2 and 3. The variance of the extinction time of a family of size *n*, Var(*S*(*n*))= 1/*λ*^2^*W*^(*d*)^(*n*), *d *= 2, 3 for the quadratic and cubic BDIMs, and the coefficient of variation *s*^(*d*)^_*n *_= (*W*^(*d*)^_*n*_)^1/2^/*E*^(*d*)^_*n *_are calculated using the formulas (A.3.8). The results are shown in Figs. 9 and 10. Some numerical values of the coefficient of variation of the extinction time for different species are given in Table 4.

**Table 2:** Family evolution under the linear BDIM (*d *= 1)

	*N*	*P*^(*d*)^(1,*N*) *10^2^	*e*^(*d*)^_*N*_	*E*^(*d*)^_*N*_	*f*^(*d*)^_*N*_	*M*^(*d*)^_*N*_	*M*^(*d*)^_*N*_/*E*^(*d*)^_*N*_	*c*^(*d*)^_*du*_	*T*^(*d*) ^_*N*_
Sce	130	0.230	33206.9	2.82	32772	249.80	88.58	7.56	94.4
Dme	335	0.404	127814.	4.72	127567	206.26	43.71	11.67	120.4
Cel	662	0.498	394794.	6.61	394593	215.36	32.58	15.80	170.2
Ath	1535	0.131	2.768*10^6^	5.98	2.77*10^6^	638.27	106.73	22.50	718.1
Hsa	1151	0.166	1.555*10^6^	5.37	1.68*10^6^	468.84	87.31	24.48	573.9
Tma	97	0.039	38872.6	2.25	36306	1231.3	547.26	3.27	201.3
Mth	43	0.315	4539.9	2.03	4234	166.47	77.09	3.33	27.7
Sso	81	0.233	13281.1	2.61	12852	252.47	97.11	4.33	54.7
Bsu	124	0.212	26441.0	3.10	25969	304.97	98.38	5.09	77.6
Eco	140	0.135	34970.6	2.90	40270	431.85	148.91	5.74	123.9

For the quadratic BDIM (*d *= 2) and for the largest family of size *N *in each genome, the table shows the probability of formation *P*^(*d*)^(1,*N*), mean number of events before extinction of the largest family *e*^(*d*)^_*N*_; mean number of events before formation of the largest family from a singleton,*f*^(*d*)^_*N*_; mean times of formation *M*^(*d*)^_*N *_and extinction *E*^(*d*)^_*N *_(in 1/*λ *units); the value of coefficient *c*^(*d*)^_*du *_= *r*_*du*_*vλ*; mean times of formation *T*^(*d*)^_*N *_in Ga (10^9 ^yrs) under *r*_*du *_= 2 × 10^-8^. The model parameters were the same as for the linear model according to (2.4). Species abbreviations: Sce, *Saccharomyces cerevisiae*, Dme, *Drosophila melanogaster*, Cel, *Caenorhabditis elegans*, Ath, *Arabidopsis thaliana*, Hsa, *Homo sapiens*, Tma, *Thermotoga maritima*, Mth, *Methanothermobacter thermoautotrophicum*, Sso, *Sulfolobus solfataricus*, Bsu, *Bacillus subtilis*, Eco, *Escherichia coli*.

**Table 3:** Family evolution under the cubic BDIM (*d *= 3).

	N	*P*^(*d*)^(1,*N*)	*e*^(*d*) ^_*N*_	*E*^(*d*) ^_*N*_	*f*^(*d*) ^_*N*_	*M*^(*d*)^_*N*_	*M*^(*d*)^_*N*_/*E*^(*d*)^_*N*_	*c*^(*d*)^_*du *_= *r*_*du*_*vλ*	*T*^(*d*)^_*N*_
Sc e	130	0.105	12315.7	0.944	12306	4.60	4.84	92.46	21.3
Dme	335	0.222	60759.4	1.390	60755	2.45	1.76	549.65	67.3
Cel	662	0.283	208472	1.804	208469	2.10	1.17	2020.37	212.1
Ath	1535	0.255	1.29*10^6^	1.390	1.29*10^6^	1.93	1.39	3754.83	362.3
Hsa	1151	0.254	756242	1.291	756238	1.65	1.27	2938.07	242.4
Tma	97	0.019	9442.5	0.781	9390	24.5	31.4	18.84	23.1
Mth	43	0.061	1530.2	0.848	1514	7.85	9.24	18.26	7.2
Sso	81	0.073	4799.6	0.960	4786	7.21	7.51	36.71	13.2
Bsu	124	0.088	10265.3	1.059	10254	6.40	6.04	63.38	20.3
Eco	140	0.071	14459.9	0.957	14446	7.34	7.67	65.06	23.9

For the cubic BDIM (*d *= 3) and for the largest family of size *N *in each genome, the table shows the probability of formation *P*^(*d*)^(1,*N*), mean number of events before extinction of the largest family *e*^(*d*)^_*N*_; mean number of events before formation of the largest family from a singleton, *f*^(*d*)^_*N*_; mean times of formation *M*^(*d*)^_*N *_and extinction *E*^(*d*)^_*N *_(in 1/*λ *units); the value of coefficient *c*^(*d*)^_*du *_= *r*_*du*_*vλ*; mean times of formation *T*^(*d*)^_*N *_in Ga (10^9 ^yrs) under *r*_*du *_= 2 × 10^-8^. The model parameters were the same as for the linear model according to (2.4). Species abbreviations: Sce, *Saccharomyces cerevisiae*, Dme, *Drosophila melanogaster*, Cel, *Caenorhabditis elegans*, Ath, *Arabidopsis thaliana*, Hsa, *Homo sapiens*, Tma, *Thermotoga maritima*, Mth, *Methanothermobacter thermoautotrophicum*, Sso, *Sulfolobus solfataricus*, Bsu, *Bacillus subtilis*, Eco, *Escherichia coli*.

**Table 4:** Coefficients of variation of the extinction and formation times for the BDIMs of different degrees

	*N*	*s*^(1)^_*N*_	*σ*^(1)^_*N*_	*s*^(2)^_*N*_	*σ*^(2)^_*N*_	*s*^(3)^_*N*_	*σ*^(3)^_*N*_
Dme	335	194.11	81.79	304.96	126.90	766.29	184.70
Cel	662	413.30	195.73	460.31	277.24	481.65	391.25
Ath	1535	885.78	421.03	1016.85	583.56	1042.86	886.95
Hsa	1151	649.77	308.40	746.56	425.21	768.04	647.23

The table shows coefficient of variation *s*^(*d*)^_*N *_of extinction *time *for the largest family; coefficient of variation *σ*^(*d*)^_*N *_of formation time for the largest family; *d *= 1,2,3 for the linear, quadratic and cubic BDIM, respectively. Species abbreviations: Dme, *Drosophila melanogaster*, Cel, *Caenorhabditis elegans*, Ath, *Arabidopsis thaliana*, Hsa, *Homo sapiens*.

#### Logistic BDIM

Let us consider the logistic modification of the rational BDIM; specifically, we will examine models with the birth and death rates of the form

*λ*_*i *_= *λ*(*i *+ *a*)(*i *+ 1)^*d*-1^(1 - *i*/(*N *+ *c*)), *δ*_*i *_= *δ *(*i *+ *b*)*i*^*d*-1^(1 - (*i *- 1)/(*N *+ *c*)).     (3.2)

**Figure 11:**
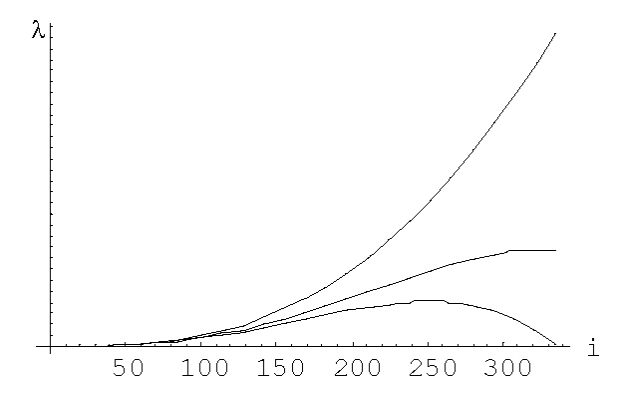
Dependence of *λ*_*i *_(3.2) at *d *= 2 on *i *with different boundary value, *c *= 1, *c *= 100, *c *= 1000 (from bottom to top). The model parameters are for *Drosophila melanogaster*.

We will refer to the parameter *c *as saturation boundary. The shape of *λ*_*i *_essentially depends on the value of *c *(Fig. 11).

The logistic model (1.1), (3.2) is a transformation (2.9) of the linear BDIM using the function:

g(*i*) = (*i *+ 1)^*d*-1^(1 - *i*/(*N *+ *c*), *c *= const ≥ 0.     (3.3)

The stationary distribution of family size frequencies for the logistic model (1.1), (3.2) is exactly the same as that for corresponding linear BDIM but the stochastic properties are different and close to the rational models, and essentially depend on the boundary *c*. With a large *c*, the model is very close to the corresponding rational model with *λ*_*i *_= *λ*(*i *+ *a*)(*i *+ 1)^*d*-1^, *δ*_*i *_= *δ *(*i *+ *b*)*i*^*d*-1^, but with small *c*, we can observe some new effects when the family size approaches *N*.

**Figure 12:**
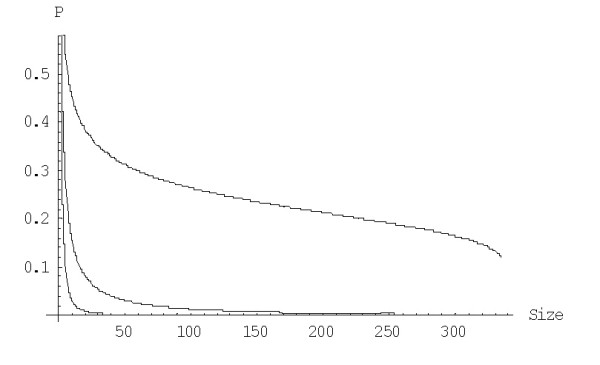
Dependence of the probability *P*^(*d*)^(1,*n*) on the family size *n *for the logistic model with *c *= 1 for *d *= 1, 2 and 3 (from bottom to top). The model parameters are for *Drosophila melanogaster*.

The probability of formation of a family of a given size from a singleton before getting to extinction for the logistic BDIM is calculated using the general formula (A.2.6) where the function *g*(*i*) is given by (3.3). The dependence of this probability on the model degree *d *under a fixed large value of the boundary *c*~*N *is similar to that for the corresponding rational models but differs under a small *c*; Fig. 12 shows this dependence for *c *= 1.

**Figure 13:**
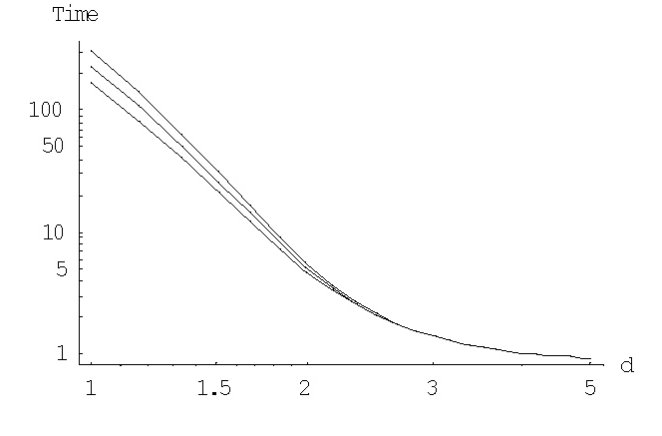
Mean time to extinction (in 1/*λ *units) of the largest families for the logistic BDIM depending on the model degree *d *for *c *= 1,*c *= 100 and *c *= 1000 (from top to bottom, in double logarithmic scale). The model parameters are for *Drosophila melanogaster*.

**Figure 14:**
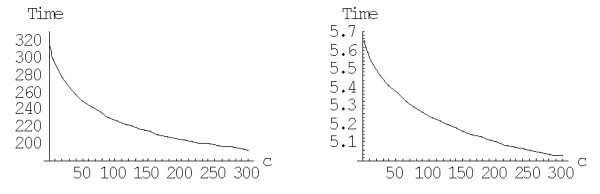
Mean time to extinction (in 1/*λ *units) of the largest families for the logistic BDIM, dependently on the boundary value *c *for *d *= 1 (left) and *d *= 2 (right). The model parameters are for *Drosophila melanogaster.*

The mean times of extinction for the logistic BDIMs are calculated using formula (A.3.4). Fig. 13 shows the mean times of extinction of the largest family, *E*^(*d*)^_*N*_, depending on the model degree *d *for different values of saturation boundary *c*. Fig. 14 shows the dependence of *E*^(*d*)^_*N *_on the saturation boundary *c *for different values of *d*.

### 4. Mean and variance of formation time for a family of the given size

Let us denote *T*(*j, n*) = inf{*t*: *X*(*t*) = *n*|*X*(0) = *j*} the time to the first passage of state *n *from the initial state *j*; *T*(*j, n*) is a random variable for each *j, n*. The mean time to the first passage for BDIM (1.1), *m*(*j, n*) = *E*(*T*(*j, n*)), can be calculated using the formula *m*(*j, n*) = *m*_0_(*j, n*) + *m*_1_(*j, n*). Here the term *m*_0_(*j*,*n*) is the mean time elapsed before the system leaves the 0 state for the last time, and the term *m*_1_(*j*,*n*) is the mean time of formation of a family of size *n *from a singleton after its last "resurrection" (see formulas (A.4.1) for details). Below we examine only the mean family formation time from an essential singleton (model (1.3)).

#### Linear BDIM

Previously, we determined the mean time of formation of a family of size *n *from a singleton for different species [43]. For the linear BDIM, the value of the mean formation time from an essential singleton is given by formula *M*^(1)^(1,*n*) = 1/*λ M*^(1)^_*n*_, where *M*^(1)^_*n*_, the mean formation time in 1/*λ *units is calculated using the formula (A.4.6)



**Figure 15:**
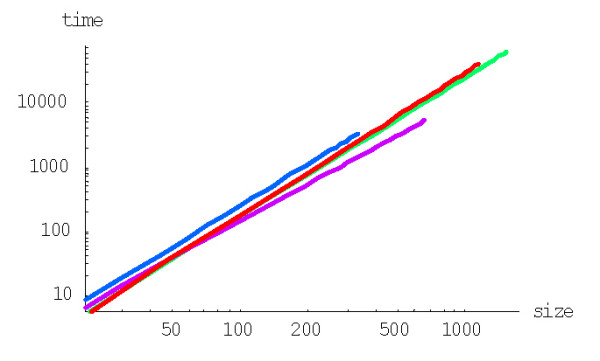
Mean time to formation (in years, Ga, with *r*_*du *_= 2*10^-8^) depending on family size for the linear BDIM (double logarithmic scale). The model parameters are for *D. melanogaster *(blue), *C. elegans *(purple), *H. Sapiens *(red), *Arabidopsis thaliana *(green).

The transition from the 1/*λ *time units to years is considered in s.6 of the Mathematical Appendix [see Additional file 1 ]. The mean formation time *E*(*T*(1, *n*)) in years is calculated using the formula (A.6.4) and the current empirical estimates of the gene duplication rate [24]. Plots of *E*(*T*(1, *n*)) for different species are shown in Fig. 15.

**Figure 16:**
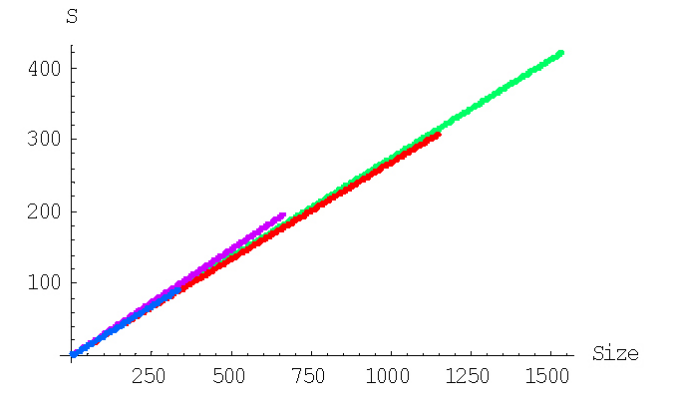
The coefficient of variation *σ*^(1)^_*n *_of family formation time depending on *n *for the linear BDIM. The model parameters are for *D. melanogaster *(blue), *C. elegans *(purple), *H. Sapiens *(red), *Arabidopsis thaliana *(green).

Once we computed the *mean *time of formation of a family of size *n *for different species, the question arises how accurately is the time *T*(*i, n*) of the first random passage through the threshold *n *predicted by the mean value. To address this problem, we estimated the variance of the time of family formation, Var(*T*(*i, n*)) using the general formulas (A.5.2) for model (1.1) and (A.5.3) for model (1.3), respectively. For the linear BDIM, the variance of the formation time for a family of size *n *from an essential singleton, *V*^(1)^_*n*_, is given by the formula (A.5.5). A more important and informative characteristic, which is independent on the model parameter *λ*, is the coefficient of variation, which is equal to . The coefficient of variation of the formation time of a family of size *n *from a singleton, *σ*^(*d*)^_*n *_= (*V*^(*d*)^_*n*_)^1/2^/*M*^(*d*)^(1;*n*) for the BDIM of degree *d *is the most relevant value. The plots of *σ*^(1)^_*n *_versus *n *for the linear model and for different species are shown in Fig. 16.

The coefficients of variation were very large for all species (see numerical values in Table 4). To summarize the results obtained for the stochastic characteristics of the linear BDIM, we found that: i) under this model, the mean time to extinction of the largest families in most genomes was much shorter than the mean time of formation of these families, and ii) using the current estimates of duplication rates in eukaryotic genomes (*r*_*du *_≈ 2 × 10^-8 ^duplications/gene/year [24]) to express the mean family formation times in real time units instead of the dimensionless 1/*λ *units, we obtain *M*^(1)^(1;*N*) ~ 10^13 ^- 10^14 ^yrs, a completely unrealistic time estimate. The mean family formation times given by the linear BDIM would become realistic only if the recent analyses underestimated the gene duplication rate by a factor of ~10^4^, which does not seem plausible. Thus, the linear BDIM cannot provide an adequate description of genome evolution, at least when only the mean time of family formation is considered. The variance of the family formation time is extremely large (the coefficient of variation is ~10^2^), and, accordingly, large deviations from the mean time, more to two orders of magnitude, are possible. However, even taking this into account, the family formation times predicted by the linear BDIM are far longer than the time allotted for life's evolution of earth. In the next section, we consider non-linear, higher order models that have the potential to yield shorter mean times of family formation.

#### Polynomial BDIMs

The mean time of formation of families from an essential singleton (or after the last "resurrection" of a family) depending on the family size *n *for the polynomial BDIMs is *E*(*T*(1,*n*)) = 1/λ M*_n _where M*_n_, the mean time of formation in 1/*λ *units can be calculated using the formulas (A.4.9)



**Figure 17:**
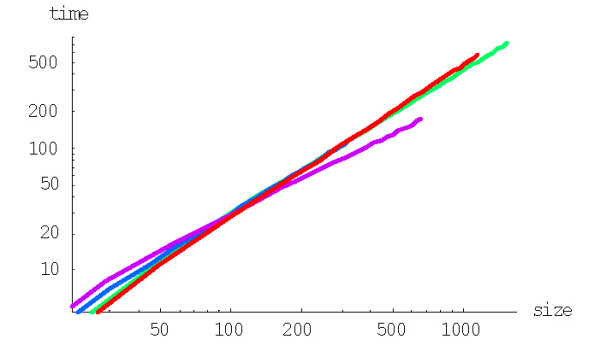
Mean time of formation (in years, Ga, with *r*_*du *_= 2*10^-8^) depending on family size *n *for the quadratic BDIM (in double logarithmic scale). The model parameters are for *D. melanogaster *(blue),*C. elegans *(purple),*H. Sapiens *(red),*Arabidopsis thaliana *(green).

Fig. 17 shows the dependence of the mean time of family formation on the family size for the quadratic BDIM in years, calculated using the formula (A.6.4). The values of mean times of formation for this BDIM are given in Table 2.

**Figure 18:**
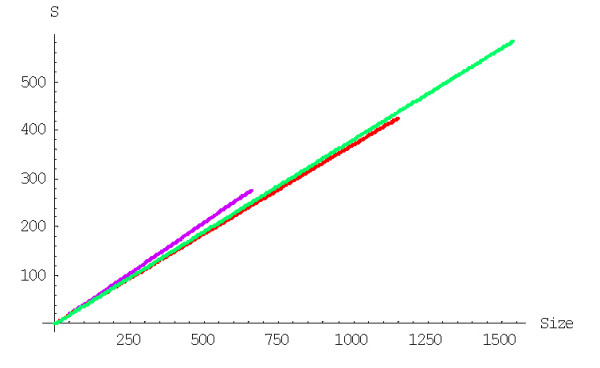
The coefficient of variation *σ*^(2)^_*n *_of formation time versus family size for the quadratic BDIM and species. The model parameters are for *D. melanogaster *(blue), *C. elegans *(purple), *H. Sapiens *(red), *Arabidopsis thaliana *(green).

The variance of formation time of a family of the size *n *can be calculated using the formula (A.5.6), with g(*j*)=*j*+1 for the quadratic BDIM and g(*j*) = (*j *+ 1)^2 ^for the cubic BDIM, respectively. The dependence of the coefficient of variation *σ*^(2)^_*n *_= (*V*^(2)^(1,*n*))^1/2^/*M*^(2)^(1;*n*) on the family size for the quadratic BDIM is shown in Fig. 18, and some numerical data are given in Table 4.

**Figure 19:**
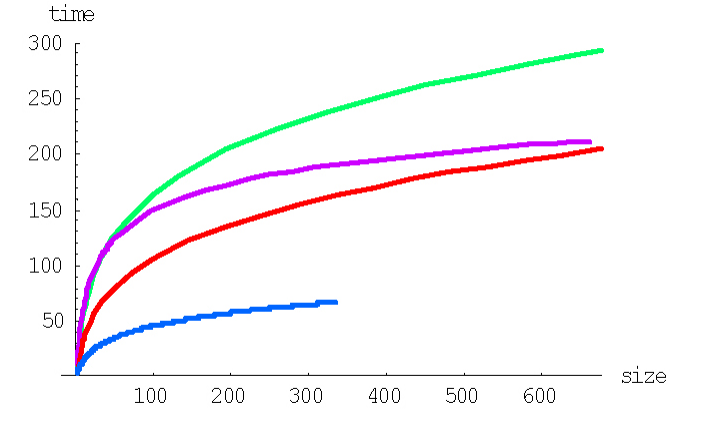
Mean time of formation (in years, Ga, with *r*_*du *_= 2*10^-8^) depending on family size *n *for the cubic BDIM. The model parameters are for *D. melanogaster *(blue), *C. elegans *(purple), *H. Sapiens *(red), *Arabidopsis thaliana *(green).

**Figure 20:**
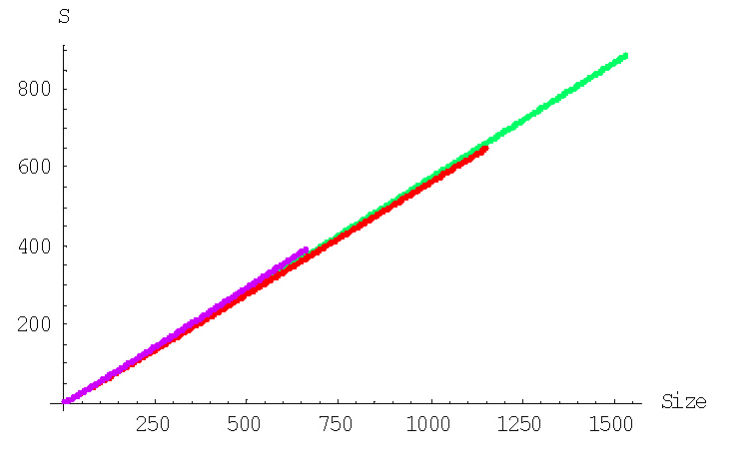
The coefficient of variation *σ*^(3)^_*n *_of formation time versus family size for the cubic BDIM. The model parameters are for *D. melanogaster *(blue), *C. elegans *(purple), *H. Sapiens *(red), *Arabidopsis thaliana *(green).

Although the variance of family formation times for the quadratic BDIM is approximately 5 orders of magnitude less than that for the linear BDIM, the values of the coefficient of variation for quadratic BDIM are about 1.3–1.5 times greater than those for the linear BDIM. Thus, the actual formation time for the largest family could differ from the mean value by several orders of magnitude with a high probability. Figures 19 and 20 show the dependence of the mean and the coefficients of variation of family formation time on family size for the cubic BDIM.

We have shown previously that the cubic model shows extremely high evolution rate comparatively with the linear and even quadratic models *under the same value of the parameter λ *[43]. On the contrary, the mean formation times in years for the quadratic and cubic models are of the same order (Tables 2 and 3). The polynomial models bring the mean time required for the formation of families of the observed size closer to realistic values but these times still remain far too long. Specifically, with the empirical estimates of the duplication rates used above for the linear BDIM, the quadratic model gives the mean family formation times ~10^11 ^yrs. This value is close to the minimum possible time of family formation that can be calculated using the duplication rate estimates of Lynch and Conery [24] and non-linear rational BDIMs.

#### Non-linear rational BDIMs

**Figure 21:**
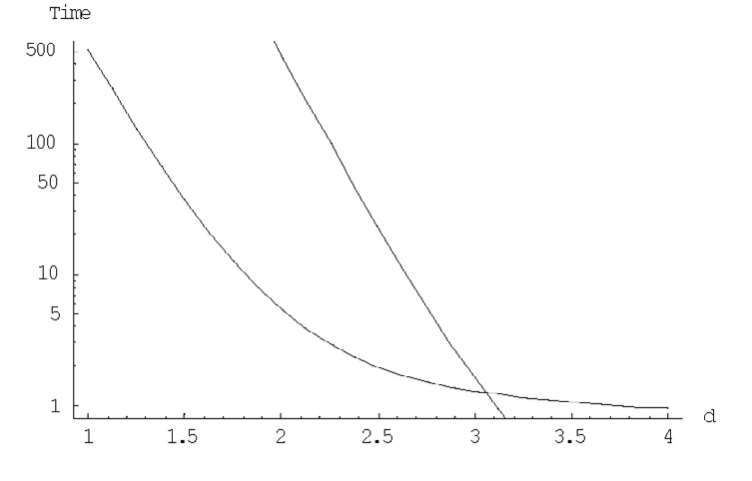
Mean times (in 1/*λ *units) of formation (upper curve *before *the point of intersection) and extinction (upper curve *after *the point of intersection) of the largest family depending on the model degree (semi-logarithmic scale). The model parameters are for *Homo sapiens.*

Let us investigate the dependence of the dynamics of the mean time of family formation on the model degree and the family size. The mean time of formation of a family of size *n *from a singleton under a fixed model degree *d, M*^(*d*)^(1;*n*), for the rational BDIM (1.1),(3.1), is calculated using the formula (A.4.9). A comparison of the mean time of formation and extinction for rational BDIMs reveals an interesting property of non-linear BDIMs: for any given family size *n*, there exists such a model degree that the times of family formation and extinction are equal (as it becomes obvious from the intersection of the respective curves in Fig. 21). Accordingly, at higher model degrees, the mean time of formation becomes shorter than the mean time to extinction. The model degree that corresponds to the point of intersection in Fig. 21 obviously depends on the size of the considered family. Tables 2 and 3 show that the mean time of formation is about 100 times more than the mean time to extinction for the largest families of different species for the quadratic BDIM and only about 10 times more for the cubic model.

**Figure 22:**
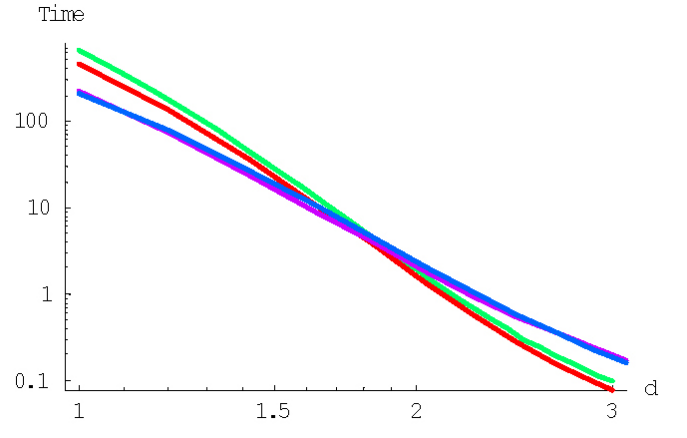
Mean time of formation of the largest family (in 1/*λ *units), *M*^(*d*)^_*N*_, for the rational BDIM depending on the model degree *d *(double logarithmic scale). The model parameters are for *D. melanogaster *(blue), *C. elegans *(purple), *H. Sapiens *(red), *Arabidopsis thaliana *(green).

**Figure 23:**
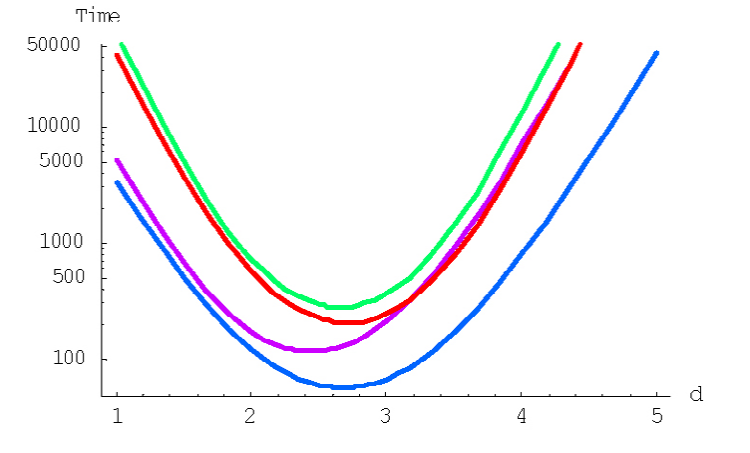
Dependence of the time (in years, Ga) required for the formation of the largest family on the model degree *d *for the rational BDIM (semi-logarithmic scale). The model parameters are for *D. melanogaster *(blue), *C. elegans *(purple), *H. Sapiens *(red), *Arabidopsis thaliana *(green).

As shown previously, increasing the degree (the "order of interaction") *d *results in indefinite decrease of the family formation time expressed in 1/*λ *time units ([43] and Fig. 22). However, we have also shown that this effect is offset by the rapid increase of the average duplication rate in the model. Assuming the gene duplication rate of ~2*10^-8 ^year^-1 ^[24], the evolution time *in years*, calculated according to the formula (A.6.5), does not decrease indefinitely, but has a minimum at the model degree between 2 and 3 (Fig. 23). Even the minimum mean time of the largest family formation achievable with the rational BDIMs is on the order of 10^11 ^years (see Table 6), which is incompatible with the age of life on Earth [43]. Thus, a rational BDIM of any degree cannot provide an adequate description of genome evolution, at least when only the mean time of family formation is considered. Accordingly, for assessing the feasibility of the formation of the largest families under a given model, the variance of the formation time should be investigated.

**Table 6:** Rational BDIM yielding the shortest mean time of family formation

	*N*	*D*	*R*^(*D*)^(*N*)	*T*^(*D*)^_*N*_
Sce	130	3.13	416.0	20.8
Dme	335	2.67	1131.0	56.55
Cel	662	2.44	2317.7	115.9
Ath	1535	2.65	5553.8	277.7
Hsa	1151	2.71	4079.5	204.
Tma	97	3.56	317.8	15.9
Mth	43	2.40	125.2	6.3
Sso	81	2.19	254.2	12.7
Bsu	124	2.05	404.4	20.
Eco	140	2.16	460.4	23.

For each genome, D is the value of the model degree *d*, which results in the minimum of the mean time of formation of the largest family, *T*^(*d*)^_*N *_= *R*^(*d*)^(*N*)/*r*_*du *_(in Ga, under indicated value of *d *and *r*_*du *_= 2 × 10^-8^) are shown. Species abbreviations: Sce, *Saccharomyces cerevisiae*, Dme, *Drosophila melanogaster*, Cel, *Caenorhabditis elegans*, Ath, *Arabidopsis thaliana*, Hsa, *Homo sapiens*, Tma, *Thermotoga maritima*, Mth, *Methanothermobacter thermoautotrophicum*, Sso, *Sulfolobus solfataricus*, Bsu, *Bacillus subtilis*, Eco, *Escherichia coli*.

Generally, the variance of the formation time of the family of the given size is given by the formulas (A.5.3) and (A.5.6). Although the variance of formation times for the quadratic and, especially, for the cubic BDIM is several orders of magnitude less than that for the linear BDIM, the coefficients of variation for both formation and extinction time increase with the model degree (Table 4). These coefficients are so large that the actual formation time of the largest family could differ from its mean value by several orders of magnitude with a high probability.

#### Logistic BDIM

**Figure 24:**
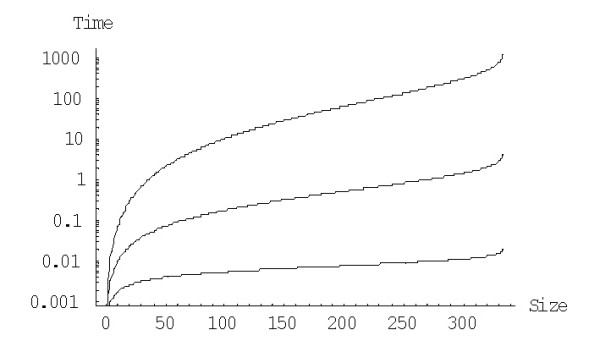
Mean time of formation (in 1/*λ *units) of a family of the given size depending on the size for the logistic BDIM with the boundary value *c *= 1 for *d *= 1, *d *= 2, *d *= 3 (from top to bottom, semi-logarithmic scale). The model parameters are for *Drosophila melanogaster.*.

**Figure 25:**
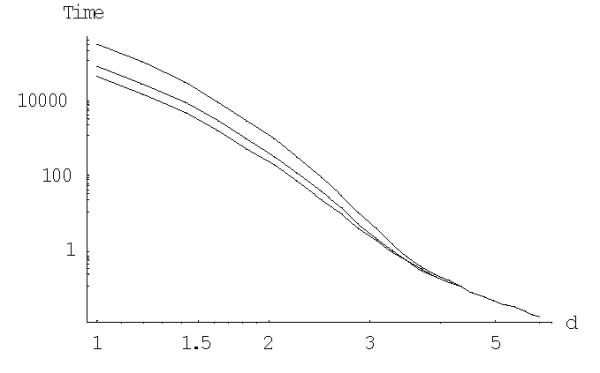
Mean time of formation (in 1/*λ *units) of the largest family for the logistic BDIM, depending on the model degree *d *for *c *= 1, *c *= 100 and *c *= 1000 (from top to bottom, double logarithmic scale). The model parameters are for *Drosophila melanogaster*.

**Figure 26:**
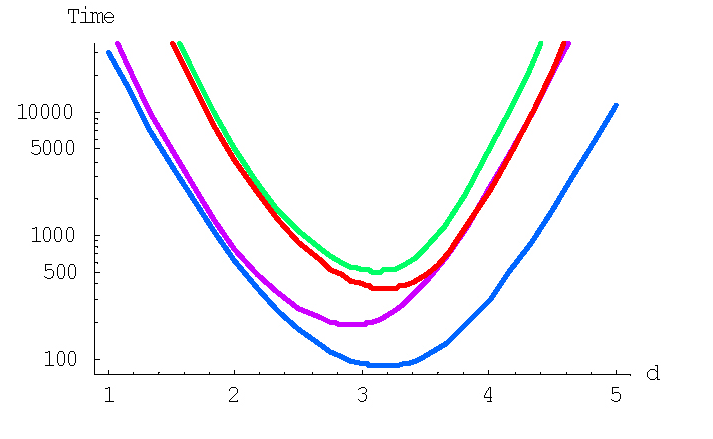
Dependence of the mean time (in years, Ga) required for the formation of the largest family for the logistic BDIM under fixed saturation boundary *c *= 1 on the model degree *d *(semi-logarithmic scale). The model parameters are for *D. melanogaster *(blue), *C. elegans *(purple), *H. Sapiens *(red), *Arabidopsis thaliana *(green).

The mean time of formation (in 1/*λ *units) of a family of size *n *from an essential singleton for the logistic BDIM (1.3), (3.2) under fixed *d *is calculated using formula (A.4.9). Fig. 24 shows the dependence of mean times of family formation, *M*^(*d*)^(1;*n*), on the family size *n *for different model degrees *d *under the fixed saturation boundary *c *= 1, and Fig. 25 shows the dependence of mean times of family formation on the boundary value (see Tables 7 and 8 for some numerical data). Similarly to the rational BDIM, increasing the degree (the "order of interaction") of the logistic model results in faster family evolution under a fixed value of the parameter *λ*. However, when this inner model parameter is excluded and the mean time of family formation is expressed *in years *according to formula (A.6.5), then we again face a restriction that does not allow indefinite shortening of the family formation time, *T*^(*d*)^_*N*_. Specifically, *T*^(*d*)^_*N *_for the logistic model with a fixed *N *has a minimum over *d*. We identified the model degrees yielding the minimum mean time of formation of the largest family for the logistic-rational BDIM. Fig. 26 and Table 9 show the dependence of *T*^(*d*)^_*N *_on *d *for the logistic model with fixed saturation boundary.

**Table 7:** Evolution of gene families under the logistic BDIM with *c *= 1 and different *d*.

	*P*^(*d*)^(1,*N*)	*E*^(*d*)^_*N*_	*M*^(*d*)^_*N*_	*M*^(*d*)^_*N*_/ *E*^(*d*)^_*N*_	*c*^(*d*)^_*du *_= *r*_*du*_*vλ*	*T*^(*d*)^_*N*_
d = 1	0.24*10^-7^	314.72	351042.	1115.4	1.7545	30795.2
d = 2	0.68*10^-3^	5.66	1247.3	220.37	10.073	628.20
d = 3	0.113	1.41	6.14	4.35	297.29	91.27

Model parameters are for *D. melanogaster*.

**Table 8:** Evolution of gene families under the logistic BDIM with *c *= 100 and different *d*.

	*P*^(*d*)^(1,*N*)	*E*^(*d*)^_*N*_	*M*^(*d*)^_*N*_	*M*^(*d*)^_*N*_/*E*^(*d*)^_*N*_	*c*^(*d*)^_*du *_= *r*_*du*_*vλ*	*T*^(*d*)^_*N*_	*L*^(*d*)^
d = 1	0.94*10^-5^	227.19	90107.4	396.62	1.7612	7934.9	32.62
d = 2	0.2*10^-2^	5.24	412.45	78.71	10.437	215.24	193.34
d = 3	0.178	1.40	3.39	2.42	354.72	25.25	6571.04

Model parameters are for *D. melanogaster*.

**Table 9:** Logistic BDIM yielding the shortest mean time of family formation under *c *= 1

	*N*	*D*	*R*^(*D*)^(*N*)	*T*^(*D*)^_*N*_
Dme	335	3.18	1726.8	86.34
Cel	662	2.92	3749.5	187.5
Ath	1535	3.11	10234.5	511.7
Has	1151	3.19	7433.9	371.7

For each genome, *D *is the value of model degree *d*, which results in the minimum of the mean time of formation of the largest family, *T*^(*d*)^_*N *_= *R*^(*d*)^(*N*)/*r*_*du *_(in Ga), is indicated. Species abbreviations: Dme, *Drosophila melanogaster*, Cel, *Caenorhabditis elegans*, Ath, *Arabidopsis thaliana*, Hsa, *Homo sapiens*.

Thus, as in the case of rational BDIMs, increase of the degree of logistic BDIMs under a fixed value of average duplication rate *r*_*du *_cannot yield mean family formation times < 10^11 ^years. Furthermore, the "saturation effect" seen in the logistic models increases the mean time of family formation compared to the corresponding rational models (compare Tables 5 and 7).

**Table 5:** Coefficients of variation of the number of events before formation of the largest family for the BDIMs of different degrees

	*N*	*Σ*^(1)^_*N*_	*Σ*^(2)^_*N*_	*Σ*^(3)^_*N*_
Dme	335	87.00	86.60	79.91
Cel	662	177.99	168.73	154.81
Ath	1535	402.66	399.03	366.50
Hsa	1151	296.42	299.31	276.23

Coefficient of variation *Σ*^(*d*)^_*N *_of the **number of events **before formation of the largest family; *d *= 1 for the linear BDIM, *d *= 2 for the quadratic BDIM, *d *= 3 for the cubic BDIM. Species abbreviations: Dme, *Drosophila melanogaster*, Cel, *Caenorhabditis elegans*, Ath, *Arabidopsis thaliana*, Hsa, *Homo sapiens*.

### 5. The mean number of elementary events before family extinction and formation

Comparing the mean family formation and extinction times predicted by BDIMs with the actual evolutionary timescale allow us to choose the most appropriate version from the examined class of models. The *number *of elementary evolutionary events namely, duplication and deletion of domains, predicted by these models is of potential interest in itself as an approximation of an important characteristic of genome evolution.

To calculate the mean number of elementary events during evolution of gene families, we employed the so-called *embedding *chains {*Y*(*n*)} instead of the original BDIM. The embedding chain {*Y*_*n*_} for a particular BDIM is a random walk with discrete time on the same set of states and transition probabilities *p*_*i*,*i*+1 _= *β*_*i *_= *λ*_*i*_/(*λ*_*i *_+ *δ*_*i*_), *p*_*i*,*i*-1 _= *μ*_*i *_= *δ*_*i*_/(*λ*_*i *_+ *δ*_*i*_) and *p*_*ij *_= 0 for all other cases (see s.7 of Mathematical Appendix for details [see Additional file 1 ]).

**Figure 27:**
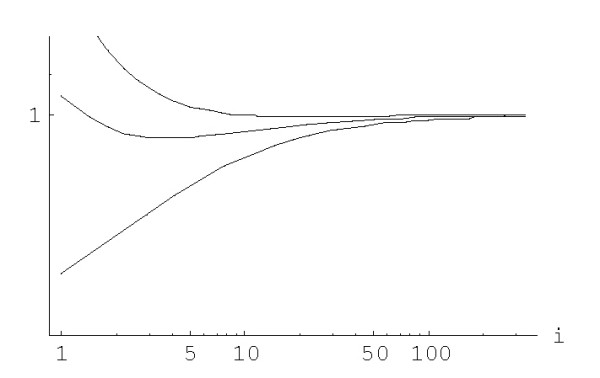
The ratio *β *_*i*_/*μ *_*i *_against family size *i *for the *rational *BDIM depending on the model degree *d*:*d *= 1, *d *= 1.6, *d *= 2 (from bottom to top), in double logarithmic scale. The model parameters are for *Drosophila melanogaster*.

**Figure 28:**
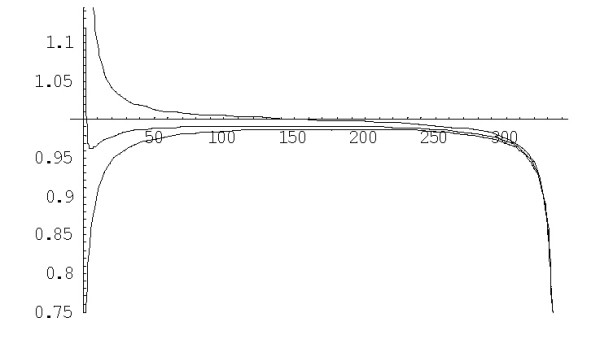
The ratio *β *_*i*_/*μ *_*i *_against family size *i *for the *logistic *BDIM (3.2) with *c *= 1 depending on the model degree *d*:*d *= 1, *d *= 1.6, *d *= 2 (from bottom to top). The model parameters are for *Drosophila melanogaster*

The transition from the state *i *to the state *i*+1 (or *i*-1) corresponds to the duplication (or deletion) of a domain in a family of size *i*. The only difference between the original birth-and-death process and the embedding chain is that the sojourn time for the embedding chain is equal to 1 for any state *i *instead of 1/(*λ*_*i *_+ *δ*_*i*_). The ratio *β*_*i*_/*μ*_*i *_(= *λ*_*i*_/*δ*_*i*_) characterizes the trend of family evolution from the state *i*, i.e., is the family more likely to grow or to shrink; for a *symmetric *random walk, *β*_*i*_/*μ*_*i *_= 1 for all *i*. The dependence of the ratio *β*_*i*_/*μ*_*i *_on *i *for different rational and logistic embedded chains is shown in Figures 27 and 28. For the rational models, *β*_*i*_/*μ*_*i *_≈ 1 for large *i*; for the logistic models, *β*_*i*_/*μ*_*i *_≈ 1 for 0 <<*i *<<*N *(however, this ratio significantly deviates from 1 at both ends of the interval of states). Thus, the behavior of the embedding chain is similar to the behavior of the symmetric random walk in the corresponding subsets of states. Informally, the plots in Figures 27 and 28 indicate that small families may preferentially grow (under higher degree models) or shrink (under low degree models) whereas the evolution of large families tends to a symmetrical random walk.

**Figure 29:**
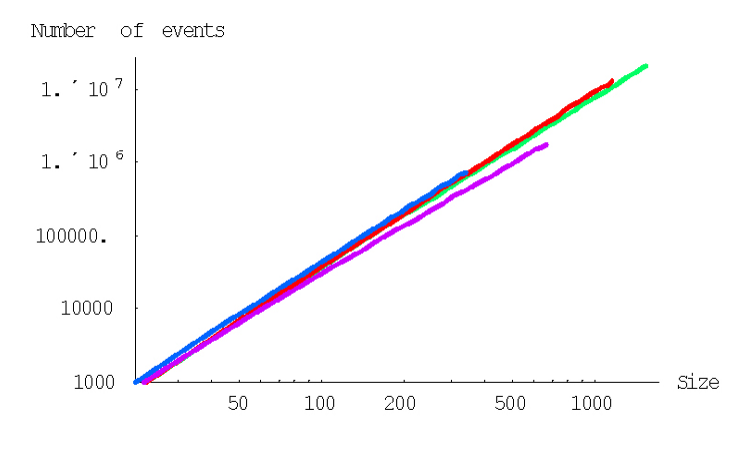
Mean number of events before the formation of a family of the given size for the linear BDIM (double logarithmic scale). The model parameters are for *D. melanogaster *(blue), *C. elegans *(purple), *H. Sapiens *(red), *Arabidopsis thaliana *(green).

**Figure 30:**
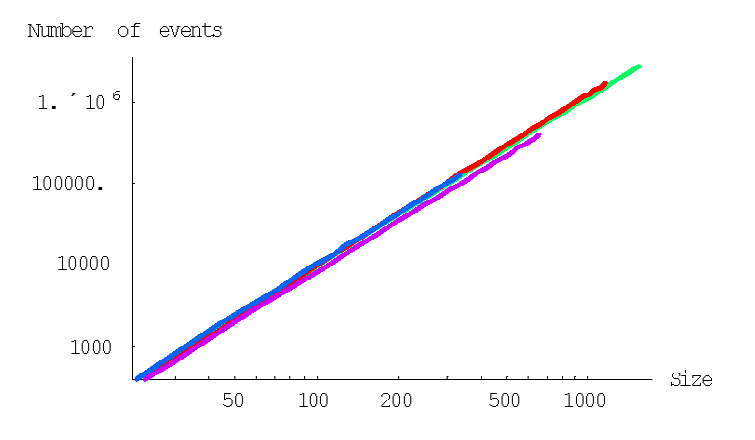
Mean number of events before the formation of a family of the given size for the quadratic BDIM (double logarithmic scale). The model parameters are for *D. melanogaster *(blue), *C. elegans *(purple), *H. Sapiens *(red), *Arabidopsis thaliana *(green).

**Figure 31:**
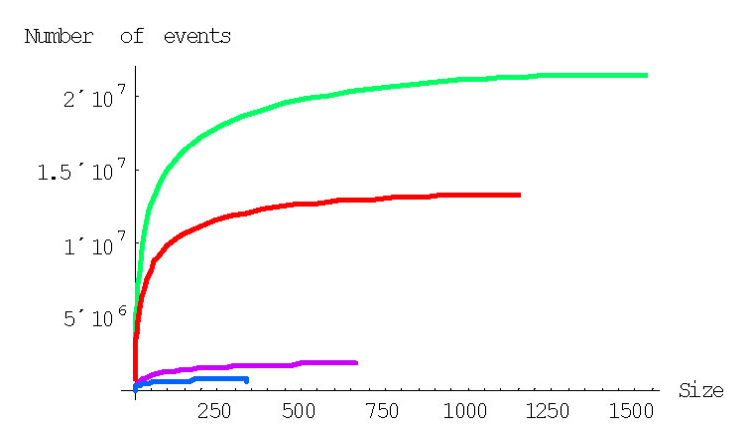
Mean number of events before extinction of a family of the given size for the linear BDIM. The model parameters are for *D. melanogaster *(blue), *C. elegans *(purple), *H. Sapiens *(red), *Arabidopsis thaliana *(green).

**Figure 32:**
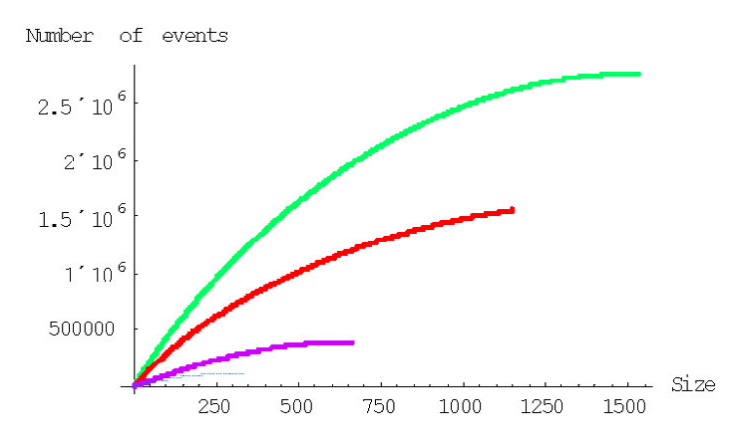
Mean number of events before extinction of a family of the given size for the quadratic BDIM. The model parameters are for *D. melanogaster *(blue), *C. elegans *(purple), *H. Sapiens *(red), *Arabidopsis thaliana *(green).

**Figure 33:**
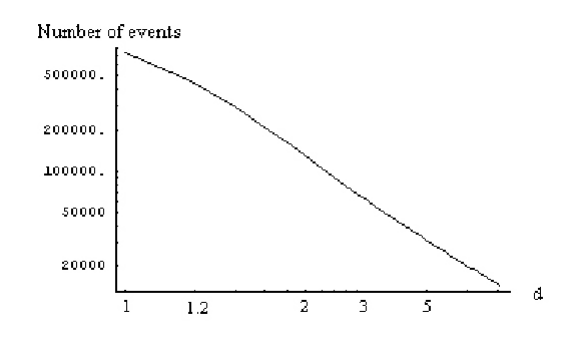
Mean number of events before the formation of the largest family against the model degree for the rational BDIM (double logarithmic scale). The model parameters are for *Drosophila melanogaster*

The mean number of elementary events before the formation of a family of the given size, *f*_*n*_, is computed using formulas (A.7.5)-(A.7.7). The plots in Figures 29 and 30 show the dependence of *f*_*n *_on the family size for different species for the linear and quadratic models, respectively. The mean number of elementary events before the extinction of a family of the given size, *e*_*n*_, is computed using formulas (A.7.13)-(A.7.15) and Figures 31 and 32 show the corresponding dependences for family extinction. Some numerical data for the mean number of elementary events for polynomial BDIMs are shown in Tables 1,2,3 and, for coefficients of variation, in Table 5. Given that all the analyzed BDIMs are balanced, i.e., the birth and death rates are asymptotically equal, it was not unexpected that the mean number of events required for the formation of a large family (or the number of events preceding the extinction of such a family) was orders of magnitude greater than the size of the family. This suggests a highly dynamic picture of genome evolution whereby numerous duplications counterbalanced by gene losses are typically involved in the evolution of large families. However, the number of events required for the formation of a family of the given size quickly drops with the increase of a model degree (Fig. 33), which may be construed as reflection of positive selection leading to amplification of family members.

### 6. Monte Carlo simulation of evolution of gene family ensembles under BDIMs of different degrees

As noticed previously [43], it is the minimum rather than the mean evolution time that is important for modeling the dynamics of evolution of genomes consisting of many gene families. Due to the large variance of the family formation time estimates (see the detailed discussion above), this value is likely to be much less than the mean. Although an analytical solution to this problem is hard to obtain, it can be examined in detail by Monte Carlo simulation analysis. As described previously [43], we employed for this analysis model parameters estimated for the human proteome. The simulated evolution started from 3000 families of size one (singletons) and continued until the largest family reached 1024 members (a convenient arbitrary number to approximate the size of the largest family in eukaryotic genomes); the simulation was run from 10 to several hundred times depending on the model degree (the time required for the simulation showed a complex, non-linear dependence on the model degree). In the course of the simulation, the number of families fluctuated due to stochastic births, deaths, and innovations of genes but, generally, tended toward the equilibrium number of ~1700, which is close to the empirically determined number of families in the human genome and is pre-determined by the choice of model parameters (the initial number of singletons did not have much impact on the model's dynamics). The time scale was adjusted such that *r*_*du *_= 2 × 10^-8 ^duplications/gene/year [24]. A series of simulations was performed for non-linear rational BDIMs with different degrees *d*.

**Figure 34:**
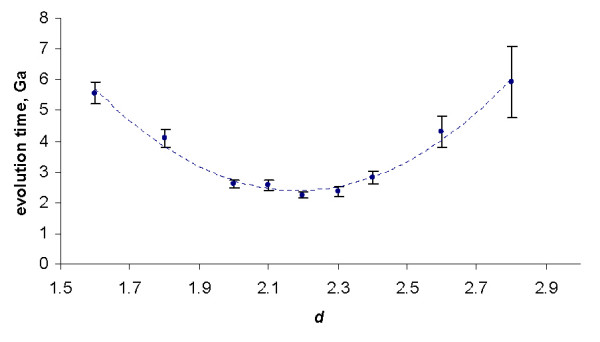
The time required for the formation of a first family with 1024 members determined by Monte Carlo simulation starting from an ensemble of 3000 singletons. The model parameters are for *Homo sapiens.*

**Figure 35:**
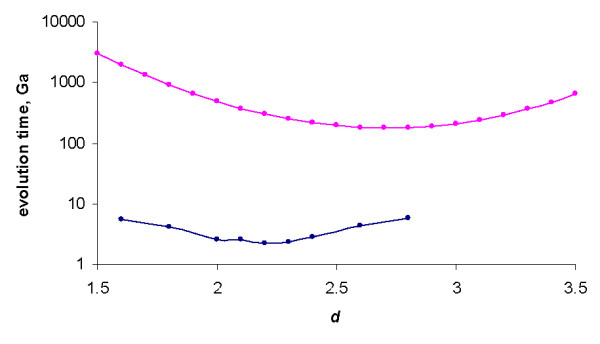
The time required for the formation of a first family with 1024 members starting from an ensemble of 3000 singletons (blue) compared to the mean time predicted by BDIMs of different orders (magenta). The model parameters are for *Homo sapiens.*

As shown in Fig. 34, the time at which the family size of 1024 members is reached for the first time depends on *d *in a similar fashion as the mean time for a single family, i.e., there is clear minimum at a particular value of *d*. At the optimal value of *d *≈ 2.2, the model reaches this family size in 2.2 ± 0.5 Ga, which is comparable to the time of evolution of eukaryotes. Compared to the minimal evolution time predicted by BDIMs of different degrees for a single family, the genome-size ensemble of gene families reached the threshold size much faster (by 1.5–2.5 orders of magnitude), and the optimum values of *d *was lower by ~0.5 (Fig. 35). The much faster formation of large families from an ensemble of singletons was predictable due to the large variation coefficient of the family formation and extinction times, but the simulation was necessary in the absence of knowledge of the exact distribution of these values.

### 7. General discussion

Here and in the previous publications [12,43,50], we describe a general class of models, which are based on the classical concept of a birth-and-death process and seem to naturally apply to the genome evolution process. Similar, although not identical and apparently less general, modeling approaches have been considered by others [6,34,51]. Even earlier, evolution of gene families has been modeled within the distinct mathematical framework of multiplicative processes [52]. The utility of birth-and-death type models in evolutionary genomics in itself is not a trivial matter but rather stems from fundamental features of genome evolution. As captured in the title of Ohno's famous book [16], although foreseen even in the early days of genetics [15,53], gene duplication probably is the principal mechanism of genome evolution. Of course, genomes cannot grow *ad infinitum *and, through most of the evolutionary history, the number of genes within a given phylogenetic lineage probably remains roughly constant. Hence duplication is intrinsically coupled to gene loss. The results of comparative genomics further show that many genes in each lineage cannot be obviously linked to other genes through duplication. Without necessarily specifying the biological mechanisms (these could involve rapid change after duplication, gene acquisition via horizontal transfer, and possibly, birth of genes from non-coding sequences), it is reasonable to view these unique genes as resulting from innovation. For genomes to maintain equilibrium, the combined rates of duplication and innovation over the entire ensemble of gene families should equal the rate of gene loss, at least when averaged over long time spans. The observed distribution of family sizes, which asymptotically tends to a power law, dictates a much more specific connection between the gene birth and death rates, namely, the second order balance. It should be noted that this form of balance does not amount to particularly fine tuning of the gene birth and death rates. The only requirement is that these rates tend to the same value when the family size tends to infinity according to the condition (1.5). In contrast, for small families, the rates may substantially differ, without significantly changing the shape of equilibrium distribution.

The incentive to examine BDIMs in detail stems from at least two fundamental questions: i) are the above elementary evolutionary mechanisms sufficient to account for the empirically observed characteristics of genomes, ii) what is the contribution of natural selection to the general, quantifiable features of genomes, such as the size distribution of gene families. The analysis of BDIMs starts to provide some answers, albeit preliminary ones. The critical observation made in the course of BDIM analysis was that different versions of these models could be readily distinguished on the basis of goodness of fit to the empirical data. This being the case, we found that the simplest possible model, in which all paralogs are considered independent, is incompatible with the data. Thus, turning to the first of the above questions, we had to conclude that, in addition to the three elementary processes, "something else" was required to model genome evolution. This "something" is the dependence or "interaction" between gene family members which results in self-accelerating family growth. In order to account for the observed stationary distribution of family sizes, it is sufficient to introduce a very weak dependence as embodied in the linear BDIM. However, when we switched from the deterministic to the stochastic version of BDIMs, which provide for the possibility of analysis of the dynamics of the systems evolution, we found that evolution under the linear BDIM was much too slow to account for the emergence of the large families of paralogs found in all genomes during the time of life's evolution. Only higher order BDIMs, with degrees between 2 and 3, i.e., with "strong interactions" between family members were found to provide for sufficiently fast evolution to be compatible with the real biological timescale.

Obviously, these findings beg the question: what is the nature of the mysterious "interactions" between paralogs? Although, on some occasions, paralogous protein do form physical complexes or interact functionally, the situation when such interaction does not exist is much more common. Therefore, the "interactions" in our models should not be perceived literally. This brings us to the second of the above major problems. BDIMs do not explicitly include the notion of selection. However, the simplest interpretation of the virtual interactions implied by the higher order BDIMs seems to be that these reflect differential tendencies of genes to form paralogous families of different sizes depending on the intensity of selection. Recent studies have shown that evolutionary fixation of gene duplications is linked to the evolutionary rates of genes. Specifically, duplications of slowly evolving genes, i.e., those that are subject to stronger purifying selection, are fixed more often [54,55]. The strong dependence of per gene duplication rates on family size in higher order BDIMs could be an abstraction of this trend. Should that be the case, we are justified to conclude that very weak selection would suffice to explain the stationary distribution of family sizes, but much stronger selective pressure is needed to account for the dynamics of genome evolution. However, the interpretation of BDIM degree as a manifestation of selection is, at this point, no more than a guess. One of the further developments of genome evolution modeling involves introducing selection explicitly and determining whether the resulting more sophisticated models will be equivalent to the higher order BDIMs explored here.

## Conclusions

In this work, we extended our analysis of stochastic Birth, Death and Innovation Models (BDIMs) of gene family evolution and showed that:

• the behavior of logistic BDIMs models, in which birth/death rates are limited for the largest families, is essentially the same as that of previously investigated BDIMs that included no such limitation

• the mean time required for the growth of large families is limited by the overall number of duplications and does not increase indefinitely with the increase of the model degree but instead passes through a minimum; even under the best-case scenario, which corresponds to a non-linear rational BDIM with *d *≈ 2.7, the mean time of the largest family formation is orders of magnitude greater than any realistic estimates based on the timescale of life's evolution;

• using the embedding chains technique, we estimated the expected number of elementary evolutionary events (gene duplications and deletions) preceding the formation of gene families of the observed size; the mean number of events exceeds the family size by orders of magnitude, suggesting a highly dynamic process of genome evolution;

• the variance of the time required for the formation of the largest families is large (coefficient of variation >> 1), which means that some families might grow much faster than the mean rate; thus, the minimal time required for family formation is more relevant for a realistic representation of genome evolution than the mean time;

• Monte Carlo simulations of family growth from an ensemble of simultaneously evolving singletons show that the time elapsed before the formation of the largest family was much shorter than the estimated mean time and approached realistic values (2.2 ± 0.5 Ga for the non-linear rational BDIM with *d *≈ 2.2).

## Contributions of individual authors

GPK developed most of the mathematical formalism and wrote the draft of the mathematical part of the manuscript; YIW performed the imitation modeling and wrote the draft of the corresponding part of the manuscript; FSB derived some of the mathematical statements; EVK contributed to the inception of the work and the formulation of the models, gave the biological interpretation of the results, wrote the background and discussion sections and extensively edited the entire manuscript.

## Supplementary Material

Additional File 1 This additional file includes proofs of some of the mathematical statements contained in the main text as well as accessory mathematical formulations. Click here for file
