# Supplementary material for: Gene family evolution: an in-depth theoretical and simulation analysis of non-linear birth-death-innovation models
Source: BMC Evol Biol. 2004 Sep 9;4:32. doi: 10.1186/1471-2148-4-32 (PMC523855; doi:10.1186/1471-2148-4-32)
Supplement: Additional File 1 — This additional file includes proofs of some of the mathematical statements contained in the main text as well as accessory mathematical formulations. [file 1471-2148-4-32-S1.pdf]

Mathematical Appendix

1. Asymptotics of ergodic distribution of the stochastic BDIM

The ergodic (or stationary) distribution (1.2) of the stochastic BDIM is completely defined by the asymptotic expansion (1.5) of the function $\chi(i)$,

$$\chi(i)=\lambda_{i-1}/\delta_i = i^s\theta(1-\gamma/i + O(1/i^2))$$

where s and γ are real numbers and θ is positive. The following main assertions describe all possible asymptotic behaviors of the stationary probabilities depending on different orders of balance in BDIM (as defined in the formulation of the theorem below).

Theorem 1. (i) *if* $s\neq 0$ *(non-balanced BDIM), then* $p_i \sim \Gamma(i)^s\theta^i i^{-\gamma}$ where $\Gamma(i)$ is the Γ-function;

(ii) *if* $s=0$ *and* $\theta\neq 1$ *(first-order balanced BDIM), then* $p_i \sim \theta^i i^{-\gamma}$;

(iii) *if* $s=0$; $\theta=1$ *and* $a\neq 0$ *(second-order balanced BDIM), then* $p_i \sim i^{-\gamma}$;

(iv) *if* $s=0$; $\theta=1$ *and* $a=0$ *(high-order balanced BDIM), then* $p_i \sim 1$.

Proof

The condition (1.5) can be rewritten as $\lambda_{i-1}/\delta_i=i^s\theta(1-\gamma/i+O(1/i^2))=i^s\theta(1-\gamma/i)(1+O(1/i^2))$.

Thus we can choose S in such a way that $\prod_{s=S}^{\infty}(1+O(1/s^2))$ converge,

$0<\prod_{s=S}^{\infty}(1+O(1/s^2))<\infty$. It follows that

$$\prod_{s=1}^{j}(\lambda_{s-1}/\delta_s) \sim \Gamma(j)^s\theta^j\prod_{s=1}^{j}(1-\gamma/s).$$

According (1.2), $p_i \sim \prod_{k=1}^{i-1}\lambda_k / \prod_{k=1}^{i}\delta_k$. So

$$p_i \sim \prod_{s=2}^{i} (\lambda_{s-1}/\delta_s) \sim \Gamma(i)^s \theta^i \prod_{s=2}^{i} (1-\gamma/s) = \Gamma(i)^s \theta^i \Gamma(i+1-\gamma)/\Gamma(i+1).$$

Applying the main asymptotic property of the Γ-function, i.e. $\Gamma(i+c)/\Gamma(i) \sim i^c$ at large i for any c, we have $\Gamma(i+1-\gamma)/\Gamma(i+1) \sim i^{-\gamma}$, and so $p_i \sim \Gamma(i)^s \theta^i i^{-\gamma}$. Q.E.D.

Corollary 1. *For a non-balanced BDIM with* $s=-1$ and $\gamma=0$, *the stationary probabilities* p_i *follow the (truncated) Poisson distribution with parameter* θ, $p_i \sim \theta^i/i!$

Corollary 2. *For a first-order balanced BDIM with* $\theta < 1$,

a) if $\gamma < 1$, *the stationary probabilities* p_i *follow the Pascal distribution with parameters* $(1-\gamma, \theta)$;

b) if $\gamma = 1$, *the stationary probabilities follow (truncated) logarithmic distribution with parameter* θ;

c) if $\gamma = 0$, *the stationary probabilities follow geometric distribution with parameter* θ.

The following implication of Theorem 1 is of principal interest.

Corollary 3. *The stationary probabilities of a BDIM have a power asymptotic behavior if and only if the BDIM is second-order balanced.*

The non-balanced BDIM (i) with $s<-1$ or $s>0$ and high-order balanced BDIM (iv) are of little practical interest because the former results in an extremely sharply dropping (or rising) distribution, whereas the latter leads to a uniform distribution of domain family sizes. Below we consider exclusively balanced BDIMs because only such models can lead to a power asymptotic of the ergodic distribution.

Transformation of BDIM

Let $g(i)$, $i=0,\ldots N$, be a positive function. Consider the new transformed model (1.1) or (1.3) under simultaneous transformation of duplication and deletion rates given by the relations:

$\lambda^*_i = \lambda_i\, g(i),\ \ \delta^*_i = \delta_i\, g(i\text{-}1).$ (A.1.1)

Then it follows from formulas (1.2) or (1.4) that the ergodic distribution for the BDIM with transformed birth and death rates, λ^*_i and δ^*_i, is the same as in the original model.

Note that the mean sojourn time in the state i of the modified model is $t^*_i = 1/(\lambda_i g(i)+\delta_i g(i\text{-}1))$. Thus, t^*_i can be arbitrarily decreased by choosing the appropriate function $g(i)$. We will see that the mean number of trials before first entrance into the state N (i.e., the mean number of duplications and deletions before the formation of the largest family) also decreases simultaneously.

Rational stochastic BDIM

Let us suppose that the birth and death rates are of the form

$$\lambda_i = \lambda\, P(i) = \lambda \prod_{k=1}^{n} (i+a_k)\wedge\alpha_k,$$

$$\delta_i = \delta\, Q(i) = \delta \prod_{k=1}^{m} (i+b_k)\wedge\beta_k$$

for $i>0$, where λ, δ are positive constants, α_k, β_k are real, and a_k, b_k are non-negative for all $k=1,\ldots N$.

The following theorem 2 and proposition 1 (proved in Ref. [1] for the deterministic BDIM) describe all possible asymptotic behaviors of the stationary probabilities of a rational BDIM. Let us denote

$$\theta = \lambda/\delta,\ \ s = \sum_{k=1}^{n} \alpha_k - \sum_{k=1}^{m} \beta_k,\ \ \gamma = \sum_{k=1}^{m} (b_k+1)\beta_k - \sum_{k=1}^{n} a_k\alpha_k.$$

Theorem 2. *The stationary probabilities of a rational BDIM have the following asymptotics*

$p_i \cong C\lambda_0\, p_0 / \lambda\, \Gamma(i)^s\, \theta^i\, i^{-\gamma}$ (A.1.2)

where the constant $C=\prod_{k=1}^{m}(\Gamma(1+b_k))^\wedge\beta_k/\prod_{k=1}^{n}\Gamma(1+a_k)^\wedge\alpha_k$.

Corollaries 1-3 are valid for a rational BDIM with the values of s and γ specified above.

The exact expressions for the stationary probabilities p_i are given in the following proposition.

Proposition 1. *For a balanced (first or higher order) rational BDIM,*

$$p_i = C\lambda_0\, p_0 /\lambda\, \theta^i \prod_{k=1}^{n} [(\Gamma(i+a_k))^\wedge\alpha_k]/\prod_{k=1}^{m} [(\Gamma(i+1+b_k))^\wedge\beta_k] \text{ for all } i=1,2,\ldots \quad \text{(A.1.3)}$$

$$p_0 =[1+ C\lambda_0/\lambda \sum_{j=1}^{N} \theta^j \prod_{k=1}^{n} (\Gamma(j+a_k))^\wedge\alpha_k/\prod_{k=1}^{m} (\Gamma(j+1+b_k))^\wedge\beta_k)]^{-1},$$

where

$$C=\prod_{k=1}^{m} [(\Gamma(1+b_k))^\wedge\beta_k]/\prod_{k=1}^{n} [\Gamma(1+a_k)^\wedge\alpha_k].$$

Polynomial stochastic BDIM

Let $\lambda_i =\lambda R(i) = \lambda\sum_{k=0}^{m} r_k\, i^{m-k}$, $\delta_i = \delta Q(i)= \delta \sum_{k=0}^{m} q_k\, i^{m-k}$

where r_k, q_k are constants and $r_0= q_0=1$. We suppose that $R(i)$, $Q(i)$ are positive for all natural i. Note that, in this case, $\chi(i) \equiv \lambda_{i-1}/\delta_i = \theta(1+(r_1 - q_1 -m)/i+O(1/i^2))$, where $\theta=\lambda/\delta$. We will suppose that $\theta\leq 1$.

According to Theorem 2, the polynomial BDIM has the stationary probabilities with *power-exponential asymptotics*

$$p_i \sim\theta^i\, i^{\rho-m} \qquad \text{(A.1.4)}$$

where $\rho = r_1 - q_1$.

In particular, if $\rho - m > -1$, the stationary probabilities p_i follow the Pascal distribution with parameters $(\rho - m + 1, \theta)$;

if $\rho - m = -1$, the stationary probabilities p_i follow the (truncated) logarithmic distribution;

if $\rho - m = 0$, the stationary probabilities p_i follow the geometric distribution;

if $\lambda = \delta$, the polynomial BDIM is second-order balanced and the stationary probabilities p_i follow the power distribution

$$p_i \sim i^{\rho - m}. \qquad \text{(A.1.5)}$$

Note that the degree of the power distribution (A.1.5) is determined only by m, the common degree of the polynomials (5.21), and ρ, the difference of the coefficients r_1 and q_1, and does not depend on other coefficients. In particular, if $r_1 = q_1$, then $p_i \sim i^{-m}$. This relation could be interpreted as follows: if the first two coefficients of polynomial rates λ_i and δ_i are equal, then the degree of the power distribution (A.1.5) is equal to the "order of interactions" of domains.

Formulas (A.1.4), (A.1.5) can be refined with the help of Proposition 2.

Proposition 2. Let $R(i) = \prod_{k=1}^{m} (i + a_k)$, $Q(i) = \prod_{k=1}^{m} (i + b_k)$. Then the stationary probabilities of the polynomial BDIM are

$$p_i = C\lambda_0\, p_0/\delta\theta^{\,i-1} \prod_{k=1}^{m} [\Gamma(i + a_k)/\Gamma(i+1+b_k)] \qquad \text{(A.1.6)}$$

where $C = \prod_{k=1}^{m} [\Gamma(1+b_k)/\Gamma(1+a_k)]$, and $p_0 = [1 + C\, \lambda_0/\lambda\, (\sum_{j=1}^{N} \theta^{\,j} \prod_{k=1}^{m} [\Gamma(j+a_k)/\Gamma(j+1+b_k)]^{-1}$.

2. Escape probabilities; probabilities of formation and extinction of families of different sizes

Let (c,d) be an interval in the space state of the Markov BDIM $X(t)$, $(c,d)=\{j: c<j<d\}$, $c\geq 0$, $d\leq N$. Let τ be the first instant of exit from the interval (c,d); let us denote $p(i;c,d)$ the probability $P_i\{X(\tau)=d\}$; verbally, $p(i;c,d)$ is the probability that the system, upon initiating its motion at the state i, will occupy the state d at the time of its first exit from the interval (c,d). Then [2, ch.3], for $c<i<d$

$$p(i;c,d)=\left(1+\sum_{j=c+1}^{i-1}\prod_{k=c+1}^{j}\delta_k/\lambda_k\right)\Big/\left(1+\sum_{j=c+1}^{d-1}\prod_{k=c+1}^{j}\delta_k/\lambda_k\right) \qquad \text{(A.2.1)}$$

In particular, the probability to reach the state n before 0 starting from the state i is

$$P(i;n)=\left(1+\sum_{j=1}^{i-1}\prod_{k=1}^{j}\delta_k/\lambda_k\right)\Big/\left(1+\sum_{j=1}^{n-1}\prod_{k=1}^{j}\delta_k/\lambda_k\right) \qquad \text{(A.2.2)}$$

and the probability to reach the state N beginning from the state 1 is

$$P(1;N)=1\Big/\left(1+\sum_{j=1}^{N-1}\prod_{k=1}^{j}\delta_k/\lambda_k\right). \qquad \text{(A.2.3)}$$

For the linear BDIM,

$$P^{(1)}(i;n)=\left(1+\frac{\Gamma(a+1)}{\Gamma(b+1)}\sum_{j=1}^{i-1}\frac{\Gamma(b+j+1)}{\Gamma(a+j+1)}\right)\Big/\left(1+\frac{\Gamma(a+1)}{\Gamma(b+1)}\sum_{j=1}^{n-1}\frac{\Gamma(b+j+1)}{\Gamma(a+j+1)}\right) \qquad \text{(A.2.4)}$$

and, after simple algebra,

$$P^{(1)}(1,n)=1\Big/\left(1+\frac{\Gamma(1+a)}{\gamma\Gamma(1+b)}\left(\frac{\Gamma(b+n+1)}{\Gamma(a+n)}-\frac{\Gamma(2+b)}{\Gamma(1+a)}\right)\right). \qquad \text{(A.2.5)}$$

where $\gamma=1+b-a$ (>0).

Note that these probabilities have the power asymptotic for large n:

$$P^{(1)}(1,n)\cong\frac{\gamma\Gamma(1+b)}{\Gamma(1+a)}\,n^{-\gamma}$$

with the same power γ as the stationary probabilities.

For the transformed BDIM,

$P^*(i;N)= (1+\sum_{j=1}^{i-1} 1/g(j)\prod_{k=1}^{j} \delta_k/\lambda_k)/(1+\sum_{j=1}^{N-1} 1/g(j)\prod_{k=1}^{j} \delta_k/\lambda_k)=$ (A.2.6)

$[1+\frac{\Gamma(a+1)}{\Gamma(b+1)}\sum_{j=1}^{i-1} (\frac{1}{g(j)}\frac{\Gamma(b+j+1)}{\Gamma(a+j+1)})] / [1+\frac{\Gamma(a+1)}{\Gamma(b+1)}\sum_{j=1}^{n-1} (\frac{1}{g(j)}\frac{\Gamma(b+j+1)}{\Gamma(a+j+1)})].$

3. The mean and variance of the time of extinction

For birth-and-death process (1.1) let us denote $S(i)= \inf\{t: X(t)=0| X(0)=i\}$, the time to the first passage of the state 0 from the initial state i; $S(i)$ is a random variable for each i. We will also refer to $S(i)$ as to the time of extinction.

Let $Z(j)= \inf\{t: X(t)=j\text{-}1| X(0)=j\}$ denote the time, starting from state j, it takes for the process to enter state j-1, and $z(j)=E(Z(j))$. Then (see, e.g., Ch. 6 in Ref. [3])

$z(j)=1/(\lambda_j+\delta_j)+ \lambda_j /(\lambda_j+\delta_j)(z(j+1)+ z(j))$,

or $\delta_j\, z(j)=1+\lambda_j\, z(j+1)$ and $z(N)= 1/\delta_N$. Hence,

$z(k)= \sum_{i=k}^{N} (\lambda_k\ldots\lambda_{i-1})/(\delta_k\ldots\delta_i)$ for $k>0$, (A.3.1)

and the mean time of extinction, $E(S(n))= \sum_{k=1}^{n} z(k)$, can be calculated using the formula

$E(S(n)) =\sum_{k=1}^{n}\sum_{i=k}^{N} (\lambda_k\ldots\lambda_{i-1})/(\delta_k\ldots\delta_i).$ (A.3.2)

In particular, for the linear 2nd order balanced BDIM

$z(k)= \frac{1}{\lambda}\frac{\Gamma(b+k)}{\Gamma(a+k)}\sum_{i=k}^{N}\frac{\Gamma(a+i)}{\Gamma(b+i+1)}$

and

$E(S(n))= \frac{1}{\lambda} E^1_n$ where

$E^{(1)}_n =\sum_{k=1}^{n}\frac{\Gamma(b+k)}{\Gamma(a+k)}\sum_{i=k}^{N}\frac{\Gamma(a+i)}{\Gamma(b+i+1)}.$ (A.3.3)

For the transformed model,

$z^*(k)= \frac{1}{\lambda}\frac{1}{g(k-1)}\frac{\Gamma(b+k)}{\Gamma(a+k)}\sum_{i=k}^{N}\frac{\Gamma(a+i)}{\Gamma(b+i+1)},$

and

$E(S(n))= \frac{1}{\lambda} E^{*}_{n}$ where

$$E^*_n=\sum_{k=1}^{n} \frac{1}{g(k-1)} \frac{\Gamma(b+k)}{\Gamma(a+k)} \sum_{i=k}^{N} [\frac{\Gamma(a+i)}{\Gamma(b+i+1)}] . \qquad (A.3.4)$$

Next, let us denote $w(j)$=Var($Z(j)$), the variance of the time to the first passage of state j-1 starting from state j. Following (Ch. 6.4 in Ref. [3]), one can get

$$w(j) =1/(\delta_j(\lambda_j +\delta_j))+ \lambda_j/\delta_j\, w(j+1) + \lambda_j/(\lambda_j +\delta_j)\, [z(j+1)+ z(j)]^2, \qquad (A.3.5)$$

$w(N)= 1/\delta_N^{\,2}$.

Let us denote

$B_j=1/(\delta_j(\lambda_j +\delta_j))+ \lambda_j/(\lambda_j +\delta_j)\, [z(j+1)+ z(j)]^2$,

so that

$w(j) =\lambda_j/\delta_j\, w(j+1) + B_j$ for $0< i<N$.

The solution of this difference equation is

$$w(j)=(1/\delta^2_N)\, (\lambda_j...\lambda_{N-1})/(\delta_j...\delta_{N-1})+ \sum_{k=j}^{N-1} B_k(\lambda_j...\lambda_{k-1})/(\delta_j...\delta_{k-1})$$

Thus the variance of the extinction time from the initial state i is

$$\mathrm{Var}(S(i))=\sum_{j=1}^{i} w(j)=$$

$$\sum_{j=1}^{i} [(1/\delta^2_N)\, (\lambda_j...\lambda_{N-1})/(\delta_j...\delta_{N-1})+ \sum_{k=j}^{N-1} B_k(\lambda_j...\lambda_{k-1})/(\delta_j...\delta_{k-1})] . \qquad (A.3.6)$$

For the linear 2nd order balanced BDIM,

$$w(j)= \frac{1}{\lambda^2} \{ \frac{1}{(b+N)^2} \frac{\Gamma(a+N)}{\Gamma(b+N)} \frac{\Gamma(b+j)}{\Gamma(a+j)} + \frac{\Gamma(b+j)}{\Gamma(a+j)} \sum_{k=j}^{N-1} B^1{}_k \frac{\Gamma(a+k)}{\Gamma(b+k)} \}$$

and so

$\mathrm{Var}(S(i))=1/\lambda^2\, W^{(1)}(i)$, where

$W^{(1)}(i)$ =

$$\frac{1}{(b+N)^2} \frac{\Gamma(a+N)}{\Gamma(b+N)} \sum_{j=1}^{i} \frac{\Gamma(b+j)}{\Gamma(a+j)} + \sum_{j=1}^{i} \frac{\Gamma(b+j)}{\Gamma(a+j)} \sum_{k=j}^{N-1} B^1{}_k \frac{\Gamma(a+k)}{\Gamma(b+k)} , \qquad (A.3.7)$$

$B^1{}_j = \frac{1}{(a+b+2j)}\{\frac{1}{(b+j)} + (a+j)\lambda^2[z(j+1)+z(j)]^2\}$.

For the transformed BDIM,

$Var(S(i)) = 1/\lambda^2\, W^*(i)$, where

$$W^*(i) = \frac{1}{g(N-1)(b+N)^2}\frac{\Gamma(a+N)}{\Gamma(b+N)}\sum_{j=1}^{i}\frac{1}{g(j)}\frac{\Gamma(b+j)}{\Gamma(a+j)} +$$

$$\sum_{j=1}^{i}\frac{\Gamma(b+j)}{\Gamma(a+j)g(j-1)}\sum_{k=j}^{N-1} B^*_k\frac{\Gamma(a+k)g(k-1)}{\Gamma(b+k)} \qquad \text{(A.3.8)}$$

where

$$B^*_j = \frac{1}{((a+j)g(j)+(b+j)g(j-1))}\{\frac{1}{(b+j)g(j-1)} + g(j)(a+j)\lambda^2[z^*(j+1)+z^*(j)]^2\}.$$

4. The mean time of the first passage for the birth-and-death process

Let us denote $T(i,n)=\inf\{t: X(t)=n| X(0)=i\}$ the time to the first passage of the state n from the initial state i; $T(i,n)$ is a random variable for each i, n. The mean time to the first passage of the state n starting from the state $j<n$, $m(j;n)=E(T(j,n))$, is calculated as follows (Ch. 3 in Ref. [2], Ch.6 in Ref. [3]).

Let $U(j)= \inf\{t: X(t)=j+1| X(0)=j\}$ be the time it takes for the process to enter state $j+1$, starting from state j. Then $T(j,n)= U(j)+T(j+1,n)$ for $j<n$ and so $T(j,n)=\sum_{k=j}^{n-1} U(k)$ where the random variables $U(k)$ are independent of each other.

Let us denote $u(j)= E(U(j))$, then

$u(j)= 1/\lambda_0(\delta_1...\delta_j)/(\lambda_1...\lambda_j) + \sum_{i=1}^{j}(\delta_{i+1}...\delta_j)/(\lambda_i...\lambda_j)$ for $j>0$,

$m(j;n)= m_0(j,n) + m_1(j,n)$, where (A.4.1)

$m_0(j,n)= 1/\lambda_0\sum_{k=j}^{n-1}(\delta_1...\delta_k)/(\lambda_1...\lambda_k)$,

$m_1(j,n)= \sum_{k=j}^{n-1}\sum_{i=1}^{k}(\delta_{i+1}...\delta_k)/(\lambda_i...\lambda_k)$ for $0<j<N$.

By the same way, for process (1.3) (no 0-state),

$$u(j)= E(U(j))= \sum_{i=1}^{j} (\delta_{i+1}...\delta_j)/(\lambda_i...\lambda_j) \quad (A.4.2)$$

and the mean time to the first passage of the state n from the state j, $M(j,n)$, is

$$M(j;n) =\sum_{k=j}^{n-1} u(j)= \sum_{k=j}^{n-1} \sum_{i=1}^{k} (\delta_{i+1}...\delta_k)/(\lambda_i...\lambda_k). \quad (A.4.3)$$

For the linear BDIM (1.1)

$E(T(j,n))= m^{(1)}(j;n)=$

$$1/\lambda_0(\frac{\Gamma(a+1)}{\Gamma(b+1)}\sum_{k=j}^{n-1}\frac{\Gamma(b+k+1)}{\Gamma(a+k+1)})+1/\lambda\sum_{k=j}^{n-1}(\frac{\Gamma(b+k+1)}{\Gamma(a+k+1)}\sum_{i=1}^{k}\frac{\Gamma(a+i)}{\Gamma(b+i+1)}). \quad (A.4.4)$$

For the linear BDIM (1.3)

$$E(T(j,n))=M^{(1)}(j;n)= 1/\lambda \sum_{k=j}^{n-1}\frac{\Gamma(b+k+1)}{\Gamma(a+k+1)}\sum_{i=1}^{k}\frac{\Gamma(a+i)}{\Gamma(b+i+1)}. \quad (A.4.5)$$

In particular, for j=1

$M^{(1)}(1;n) =1/\lambda\ M^{(1)}{}_n$ where

$$M^{(1)}{}_n =\sum_{k=1}^{n-1}\frac{\Gamma(b+k+1)}{\Gamma(a+k+1)}\sum_{i=1}^{k}\frac{\Gamma(a+i)}{\Gamma(b+i+1)}. \quad (A.4.6)$$

For the transformed model (1.1),

$$m^*(j;n)=1/\lambda_0(\frac{\Gamma(a+1)}{\Gamma(b+1)}\sum_{k=j}^{n-1}\frac{1}{g(k)}\frac{\Gamma(b+k+1)}{\Gamma(a+k+1)}+$$

$$1/\lambda\sum_{k=j}^{n-1}\frac{1}{g(k)}\frac{\Gamma(b+k+1)}{\Gamma(a+k+1)}\sum_{i=1}^{k}\frac{\Gamma(a+i)}{\Gamma(b+i+1)}. \quad (A.4.7)$$

For the transformed model (1.3),

$E(T(j,n))= M^*(j;n)$ where

$$M^*(j;n) =1/\lambda\sum_{k=j}^{n-1}\frac{1}{g(k)}\frac{\Gamma(b+k+1)}{\Gamma(a+k+1)}\sum_{i=1}^{k}\frac{\Gamma(a+i)}{\Gamma(b+i+1)}. \quad (A.4.8)$$

In particular, for j=1

$M^*(1;n) =1/\lambda\ M^*{}_n$ where

$$M^*{}_n =\sum_{k=1}^{n-1}\frac{1}{g(k)}\frac{\Gamma(b+k+1)}{\Gamma(a+k+1)}\sum_{i=1}^{k}\frac{\Gamma(a+i)}{\Gamma(b+i+1)}. \quad (A.4.9)$$

5. The variance of the first passage time for the birth-and-death process

The variance of the first passage time is also of interest in many problems (see, e.g., s.7.3 in Ref. [4]) for finite Markov chains with discrete time and Ch. 6 in Ref. [3] for continuous birth-and-death process). Let us denote $v(j)$= Var($U(j)$), the variance of the time to the first passage of state j+1 starting from the state j. Then (see Ch. 6 in Ref. [3]):

$$v(j)= (1/\lambda_0)^2(\delta_1\ldots\delta_j)/(\lambda_1\ldots\lambda_j) + \sum_{i=1}^{j} A_i(\delta_{i+1}\ldots\delta_j)/(\lambda_{i+1}\ldots\lambda_j) \text{ for } j>0, \qquad \text{(A.5.1)}$$

$$v(0)= (1/\lambda_0)^2,$$

where

$A_j=1/(\lambda_j(\lambda_j+\delta_j))+ \delta_j/(\lambda_j+\delta_j)\,[u(j-1)+ u(j)]^2$, $u(j)$ is given in (A.4.2). So

$$\mathrm{Var}(T(i,n))=\sum_{j=i}^{n-1} v(j)=$$

$$\sum_{j=i}^{n-1} [(1/\lambda_0)^2 (\delta_1\ldots\delta_j)/(\lambda_1\ldots\lambda_j) + \sum_{k=1}^{j} A_k(\delta_{k+1}\ldots\delta_j)/(\lambda_{k+1}\ldots\lambda_j)]. \qquad \text{(A.5.2)}$$

Similarly, for model (1.3) (no 0-state), the variance of the time to the first passage of state j+1 starting from state j

$$V(j)= (1/\lambda_1)^2 (\delta_2\ldots\delta_j)/(\lambda_2\ldots\lambda_j) + \sum_{i=2}^{j} A_i(\delta_{i+1}\ldots\delta_j)/(\lambda_{i+1}\ldots\lambda_j) \text{ for } j>1,\ \ V(1)= 1/\lambda^2{}_1.$$

So for model (1.3) the variance of the time to the first passage of the state n from initial state i, is

$$Var(T(i,n)) =\sum_{j=i}^{n-1} \{(1/\lambda_1)^2 (\delta_2\ldots\delta_j)/(\lambda_2\ldots\lambda_j)+ \sum_{k=2}^{j} A_k(\delta_{k+1}\ldots\delta_j)/(\lambda_{k+1}\ldots\lambda_j)\}. \qquad \text{(A.5.3)}$$

The most important specific case is the variance of $T(1,n)$:

$$V_n \equiv Var(T(1,n)) = \sum_{j=1}^{n-1} [1/\lambda^2{}_1(\delta_2\ldots\delta_j)/(\lambda_2\ldots\lambda_j)+ \sum_{k=2}^{j} A_k(\delta_{k+1}\ldots\delta_j)/(\lambda_{k+1}\ldots\lambda_j)]. \qquad \text{(A.5.4)}$$

For the linear BDIM (1.3), (2.1)

$Var(T(1,n)) = \dfrac{1}{\lambda^2} V^{(1)}{}_n$ where

$$V^{(1)}{}_n= \frac{1}{(1+a)^2}\frac{\Gamma(a+2)}{\Gamma(b+2)}\sum_{j=1}^{n-1}\frac{\Gamma(b+j+1)}{\Gamma(a+j+1)} + \qquad \text{(A.5.5)}$$

$$\sum_{j=1}^{n-1}\frac{\Gamma(b+j+1)}{\Gamma(a+j+1)}\sum_{k=2}^{j} A_k \frac{\Gamma(a+k+1)}{\Gamma(b+k+1)},$$

$A^1_j = \frac{1}{(a+b+2j)} \{ \frac{1}{a+j} + (b+j)\, \lambda^2\, [u(j\text{-}1) + u(j)]^2 \}.$

For the transformed BDIM,

$$V^*_n = \frac{1}{g(1)((1+a)\lambda)\hat{}2} \frac{\Gamma(a+2)}{\Gamma(b+2)} \sum_{j=1}^{n-1} \frac{1}{g(j)} \frac{\Gamma(b+j+1)}{\Gamma(a+j+1)} + \qquad \text{(A.5.6)}$$

$$\sum_{j=1}^{n-1} \frac{1}{g(j)} \frac{\Gamma(b+j+1)}{\Gamma(a+j+1)} \sum_{k=2}^{j} g(k) A^*_k \frac{\Gamma(a+k+1)}{\Gamma(b+k+1)},$$

where

$$A^*_j = \frac{1}{(a+j)g(j)+(b+j)g(j-1)} \{ \frac{1}{(a+j)g(j)} + (b+j)g(j-1)\, \lambda^2\, [u(j\text{-}1) + u(j)]^2 \}.$$

6. The average rate of duplication and the mean time of formation of families

By definition, the mean duplication rate per domain is:

$$r_{du} = \sum_{i=1}^{N-1} p_i \lambda_i / i\, . \qquad \text{(A.6.1)}$$

Let us introduce the coefficient

$c_{du} = r_{du}/\lambda,$

which connects the empirical value of r_{du} with the model parameter λ. Then, for the rational model (1.1), (3.1) of degree d

$$c_{du}(d,N) = (\sum_{i=1}^{N-1} p_i \lambda_i / i)/\lambda = p_1 (\sum_{i=1}^{N-1} \frac{\Gamma(2+b)}{i\Gamma(1+a)} \frac{\Gamma(i+a+1)}{\Gamma(i+1+b)} (i+1)^{d\text{-}1}) =$$

$$\sum_{i=1}^{N-1} \frac{\Gamma(i+a+1)}{i\Gamma(i+1+b)} (i+1)^{d\text{-}1} / \sum_{i=1}^{N} \frac{\Gamma(a+i)}{\Gamma(b+i+1)}\, . \qquad \text{(A.6.2)}$$

According to (A.4.8), the mean time of formation of a family of size n from an essential singleton (for which extinction is not allowed) for this BDIM is

$$E(T(1,n)) = 1/\lambda\, M^d(1;n) = \qquad \text{(A.6.3)}$$

$$1/\lambda \sum_{m=1}^{n-1} \left(\frac{\Gamma(b+m+1)}{(m+1)^{d-1}\Gamma(a+m+1)} \sum_{k=1}^{m} \frac{\Gamma(a+k)}{\Gamma(b+k+1)} \right).$$

Then, excluding the model parameter λ from (A.6.3) using the relation $1/\lambda= c_{du}(d,N)/ r_{du}$, we obtain the following expression for the mean time of family formation, which is expressed through the mean duplication rate r_{du}

$$E(T(1,n))= c_{du}(d,N)M^{d}(1; n)/r_{du} . \qquad \text{(A.6.4)}$$

Finally, the mean time of formation of the largest family of the size N in years, $T^{(d)}_N$, is

$$T^{(d)}_N =E(T(1,N))= R^{(d)}(N)/r_{du} , \qquad \text{(A.6.5)}$$

where

$$R^{(d)}(N) =\left[\sum_{i=1}^{N-1} \frac{\Gamma(i+a+1)}{i\Gamma(i+1+b)} (i+1)^{d-1} / \sum_{i=1}^{N} \frac{\Gamma(a+i)}{\Gamma(b+i+1)} \right] *$$

$$\sum_{m=1}^{N-1} \left(\frac{\Gamma(b+m+1)}{(m+1)^{d-1}\Gamma(a+m+1)} \sum_{k=1}^{m} \frac{\Gamma(a+k)}{\Gamma(b+k+1)} \right).$$

7. The mean and variance of the number of events before formation and extinction of the largest family

To calculate the mean number of elementary events of genome evolution, i.e., deletions and duplications, we use the so-called *embedding* birth-and-death chain $\{Y(n)\}$instead of the initial BDIM. For a given Markov birth-and-death process $\{X(t)\}$ with continuous time, the embedding chain $\{Y_n\}$ is, by definition, the Markov chain with discrete time on the same set of states with the transition probabilities $\{p_{ij}\}$:

$p_{i,i+1} =\beta_i =\lambda_i /(\lambda_i +\delta_i)$, $p_{i,i-1} =\mu_i =\delta_i /(\lambda_i +\delta_i)$ and p_{ij} =0 for all other cases. (A.7.1)

Many problems bearing on the process $X(t)$ can be solved by analyzing the embedding Markov chain $\{Y_n\}$, e.g., the probability of arriving at one state before another, the probability of ever reaching a given state, escape probabilities, the mean number of trials before extinction, etc. Let us emphasize that, for the chain Y_n, the sojourn time in every state is equal to 1 and hence its extinction time is equal to the number of elementary events (deletions and duplications) before the extinction of a family of the given size.

Similarly, the first passage time is equal to the number of elementary events, which are necessary for the formation of a family of the given size from a singleton.

The problem of the mean number of transitions from one state to another for birth-and-death chains is readily solved (see, e.g., Ch. 3 in Ref. [2]). Let us note that all formulas for the mean time of extinction or formation for the original BDIM (see sections A3, A4 of Mathematical Appendix) could be applied for computing the mean number of transitions if the intensities of transitions, λ_i and δ_i, are replaced by the probabilities of transitions for the corresponding embedding chain, $\beta_i=\lambda_i(\lambda_i+\delta_i)$ and $\mu_i=\delta_i(\lambda_i+\delta_i)$.

In more detail, let us denote $F(i,n)=\inf\{k: Y(k)=n| Y(0)=i\}$ the number of transitions before the first passage of the state n starting from state i.
Let $h(j)= \inf\{k: X(k)=j+1| X(0)=j\}$ be the number of steps it takes for the chain to enter state j+1, starting from state j. Then $F(j,n)= h(j)+F(j+1,n)$ for $j<n$ and so $F(j,n)=\sum_{k=j}^{n-1} h(k)$ where the random variables $h(k)$ are independent each other. Using the Markov property, we can write:

$$E[h(j)|I_j]=1+(1- I_j)(E[h(j-1)]- E[h(j)]), \qquad \text{(A.7.2)}$$

where I_j=1 if the first transition from j is to j+1, and I_j=0 otherwise. So,

$E(h(j))=1+\beta_j(E(h(j-1)) + E(h(j)))$,

and

$E(h(j))=1/\alpha_j+\beta_j/\alpha_j\, E(h(j-1))$. Hence

$$E(h(j)) =\sum_{i=1}^{j} (\beta_{i+1}\ldots\beta_j)/(\alpha_{i}\ldots\alpha_j)= \sum_{i=1}^{j} (1+\delta_i/\lambda_i)(\delta_{i+1}\ldots\delta_j)/(\lambda_{i+1}\ldots\lambda_j) \qquad \text{(A.7.3)}$$

and the mean number of transitions before the first passage of the state n starting from state j is

$$EF(j;n) = \sum_{k=j}^{n-1} \sum_{i=1}^{k} (1+\delta_i/\lambda_i) (\delta_{i+1}\ldots\delta_k)/(\lambda_{i+1}\ldots\lambda_k). \qquad \text{(A.7.4)}$$

So the mean number of events before the formation of a family of the largest size from an essential singleton, f_N, is

$$f_N \equiv EF(1;n) = \sum_{k=1}^{N-1} \sum_{i=1}^{k} (1 + \delta_i/\lambda_i)(\delta_{i+1}\ldots\delta_k)/(\lambda_{i+1}\ldots\lambda_k). \quad \text{(A.7.5)}$$

For the linear balanced BDIM,

$$f^{(1)}_N = \sum_{k=1}^{N-1} \frac{\Gamma(b+k+1)}{\Gamma(a+k+1)} \sum_{i=1}^{k} (1+\frac{i+b}{i+a})\frac{\Gamma(a+i+1)}{\Gamma(b+i+1)}. \quad \text{(A.7.6)}$$

For the transformed model,

$$f^*_N = \sum_{k=1}^{N-1} \frac{\Gamma(b+k+1)}{g(k)\Gamma(a+k+1)} \sum_{i=1}^{k} (g(i)+\frac{i+b}{i+a}\, g(i\text{-}1))\frac{\Gamma(a+i+1)}{\Gamma(b+i+1)}. \quad \text{(A.7.7)}$$

To compute the variance of the number of transitions before the first passage of the state n starting from state j (see Ch. 6.4 in Ref. [3]), let us note that it follows from (A.7.2) that $Var(E[h(j)|I_j]) = (E[h(j\text{-}1)] + E[h(j)])^2\, Var(I_j) = (E[h(j\text{-}1)] + E[h(j)])^2(\lambda_j\delta_j)/(\lambda_j+\delta_j)^2$.

Similarly,

$Var(h(j)|I_j) = (1 - I_j)[Var(h(j\text{-}1)) + Var(h(j))]$, and so,

$E[Var(h(j)|I_j)] = \delta_j/(\lambda_j+\delta_j)\ [Var(h(j\text{-}1)) + Var(h(j))]$.

According to the conditional variance formula, $Var(X \mid I) = Var(E[X|I]) + E[Var(X)|I)]$, the variance of the number of steps before the first passage of the state j+1 from the state j is

$Var(h(j)) = (E[h(j\text{-}1)] + E[h(j)])^2(\lambda_j\delta_j)/(\lambda_j+\delta_j)^2 + \delta_j/(\lambda_j+\delta_j)\ [Var(h(j\text{-}1)) + Var(h(j))]$, or

$$Var(h(j)) = (E[h(j\text{-}1)] + E[h(j)])^2\delta_j/(\lambda_j + \delta_j) + \delta_j/\lambda_j\ [Var(h(j\text{-}1)) \quad \text{(A.7.8)}$$

for j>1, $Var(h(1)) = 0$.

So

$Var(h(j)) = C_j + \delta_j/\lambda_j\ [Var(h(j\text{-}1))$ where

$$C_j = (E[h(j\text{-}1)] + E[h(j)])^2\delta_j/(\lambda_j + \delta_j), \quad \text{(A.7.9)}$$

and hence

$$Var(h(j)) = \sum_{k=2}^{j} C_k(\delta_{k+1}\ldots\delta_j)/(\lambda_{k+1}\ldots\lambda_j)\}\ \text{for } j>1.$$

Finally, the variance of the number of transitions before the first passage of state n from the initial state 1 is

$Var(F(1,n)) = \sum_{j=1}^{n-1} \sum_{k=2}^{j} C_k(\delta_{k+1}\ldots\delta_j)/(\lambda_{k+1}\ldots\lambda_j)\}$ for $n>2$, (A.7.10)

$Var(F(1,2)) = 0$.

For the linear BDIM (2.3),

$$Var(F(1,n)) = \sum_{j=1}^{n-1} \frac{\Gamma(b+j+1)}{\Gamma(a+j+1)} \sum_{k=2}^{j} C^1{}_k \frac{\Gamma(a+k+1)}{\Gamma(b+k+1)}, \quad \text{(A.7.11)}$$

where $C^1{}_k = \frac{b+k}{a+b+2k}(E[h(j\text{-}1)] + E[h(j)])^2$,

$E[h(j)]$ is computed for the linear model by (A.7.3).

For the transformed BDIM,

$$Var(F^*(1,n)) = \sum_{j=1}^{n-1} \sum_{k=2}^{j} C^*{}_k(\delta_{k+1}\ldots\delta_j)/(\lambda_{k+1}\ldots\lambda_j)\} =$$

$$\sum_{j=1}^{n-1} \frac{\Gamma(b+j+1)}{g(j)\Gamma(a+j+1)} \sum_{k=2}^{j} C^*{}_k \frac{g(k)\Gamma(a+k+1)}{\Gamma(b+k+1)}, \quad \text{(A.7.12)}$$

where

$$C^*{}_k = (E[h^*(k\text{-}1)] + E[h^*(k)])^2 \frac{(b+k)g(k-1)}{(a+k)g(k)+(b+k)g(k-1)},$$

$E[h^*(j)]$ is computed for the transformed model by (A.7.3).

Similarly, the mean number of events before extinction of a family of size n, e_n, is

$$e_n = E(S(n)) = \sum_{k=1}^{n} \sum_{i=k}^{N} (\alpha_k\ldots\alpha_{i-1})/(\beta_k\ldots\beta_i) =$$

$$\sum_{k=1}^{n} \sum_{i=k}^{N} (1+\lambda_i/\delta_i) \prod_{j=k}^{i-1} (\delta_j/\lambda_j) \quad \text{(A.7.13)}$$

(taking into account that $\beta_N=1$).

For the linear balanced BDIM,

$$e^{(1)}{}_n = \sum_{k=1}^{n} \left[\frac{\Gamma(N+b)\Gamma(k+a)}{\Gamma(N+a)\Gamma(k+b)} + \sum_{i=k}^{N-1} \frac{\Gamma(i+b)\Gamma(k+a)}{\Gamma(i+a)\Gamma(k+b)}\left(1+\frac{i+a}{i+b}\right)\right]. \quad \text{(A.7.14)}$$

For the transformed model,

$$e^*_n = \sum_{k=1}^{n} \left[\frac{g(k-1)}{g(N-1)} \frac{\Gamma(N+b)\Gamma(k+a)}{\Gamma(N+a)\Gamma(k+b)} + \right.$$

$$\left. \sum_{i=k}^{N-1} \frac{g(k-1)}{g(i-1)} \frac{\Gamma(i+b)\Gamma(k+a)}{\Gamma(i+a)\Gamma(k+b)} \left(1 + \frac{(i+a)g(i)}{(i+b)g(i-1)}\right) \right] \qquad \text{(A.7.15)}$$

References

1. Karev GP, Wolf YI, Rzhetsky AY, Berezovskaya FS, Koonin EV: **Birth and death of protein domains: A simple model of evolution explains power law behavior**. *BMC Evolutionary Biology* 2002 2:18.
2. Bhattacharya R, Waymire E: *Stochastic processes with applications*. New York: Wiley; 1990.
3. Ross SM: *Introduction to probability models*. Boston: Academic Press; 1989.
4. Hunter JJ: *Mathematical techniques of applied probability*. New York: Academic Press; 1983.
